# The Barriers and Facilitators Influencing Nurses' Political Participation or Healthcare Policy Intervention: A Systematic Review and Qualitative Meta-Synthesis

**DOI:** 10.1155/2024/2606855

**Published:** 2024-06-28

**Authors:** Nam Kyung Han, Gwang Suk Kim

**Affiliations:** ^1^Department of Nursing, Gyeongbuk College of Health, Daehakro 168 Gimcheon City, Gyeongbuk 39525, Republic of Korea; ^2^Mo-im Kim Nursing Research Institute, College of Nursing, Yonsei University 50-1 Yonsei-ro, Seodaemun-gu, Seoul 03722, Republic of Korea

## Abstract

**Background:**

Nurses, who comprise the largest proportion of healthcare professionals, must advocate for public health in a changing healthcare environment. Therefore, nurses have a social responsibility to be interested in politics, political participation, or healthcare policy interventions as leaders in healthcare policy reforms. However, previous research has reported that nurses' political interests and participation are insufficient in most countries.

**Aim:**

This study systematically reviewed and synthesized qualitative data to identify the barriers and facilitators influencing nurses' political participation and healthcare policy interventions.

**Methods:**

This study performed a systematic review and qualitative meta-synthesis. Literature searches were conducted using seven databases to comprehensively examine published journals, including doctoral dissertations, until December 31, 2023. The selection criteria for this study were articles analyzed using phenomenology, ethnography, qualitative research, and grounded theory, targeting nurses with extensive experience in healthcare policy intervention and political activities. Two researchers, professors in nursing with extensive experience in healthcare policy interventions and qualitative research screened the qualitative studies and extracted the data. Eighteen papers were analyzed, and the quality of each study was evaluated using the Critical Appraisal Skills Program Qualitative Checklist. Meta-ethnography was applied as the qualitative meta-synthesis method using ATLAS. ti.

**Results:**

Barriers include nurses' lack of political interest and competence, nursing education, restrictive organizational cultures, and the nursing profession's political activities. Facilitators include recognizing social responsibilities, enhancing political competence, innovating organizational environments, and strengthening nursing organizations' political activities and policy interventions.

**Conclusions:**

This study could be used as data to enhance nurses' political participation and to plan policy interventions and strategies. *Implication for Nursing Management*. To activate nurses' political participation in the future, it is necessary to develop strategies, such as developing nursing political education programs and expanding opportunities for policy intervention.

## 1. Introduction

Since the United Nations (UN) introduced 17 sustainable development goals (SDGs) in 2015 [1], the World Health Organization (WHO) has encouraged countries to reform healthcare policies and achieve key health-related SDGs [2]. Furthermore, the UN and WHO emphasize the need to enhance healthcare professionals' political participation and healthcare policy intervention based on their social responsibility to accomplish the SDGs [2].

Politics is an authoritative distribution of social values [3], and healthcare professionals can contribute to the effective maintenance and development of a limited-resource healthcare system through political participation and policy interventions in policy-making processes [2]. Political participation is defined as activities that directly or indirectly influence government policies, such as election voting, election campaigns, rallies, legislative lobbying, and petitions [4]. Regarding this, 27.9 million nurses, accounting for approximately 59% of the world's largest healthcare workforce [2], must advocate for public health and nurses' rights in a changing healthcare environment, as specified by the International Council of Code of Ethics for Nurses [5]. In addition, nurses have a social responsibility to be interested in politics and to engage in political participation or policy interventions as leaders in healthcare policies [6–9]. In this regard, analytical studies have been reported in some countries, such as the United States, South Korea, and Iran, on the activities and successes of nurse political activists in contributing to the reform or introduction of healthcare policies [10–14]. However, it has been reported in many countries that nurses' political interest or participation is insufficient [6–9, 15]. Therefore, based on the experience of the political participation of skilled nurse activists, it is necessary to explore the barriers to and facilitators of political participation to strengthen the political competencies of nurses.

Although there have been a few systematic review studies related to nurses' political participation or healthcare policy intervention, there have been few meta-comprehensive studies, and they mainly analyze nurses' political competence or role, policy intervention, and policy advocacy experience [8, 15–18]. In this regard, Benton et al. [16] analyzed a mixed study on the status and results of research on nurses' political competence and policy pursuit. Etowa et al. [8] performed a systematic review of the significant involvement of nurses and midwives in policy development in low- and middle-income countries, focusing on their experiences. Hajizadeh et al. [15] conducted a systematic review identifying factors that influence nurses' participation in health policy-making, highlighting three main themes: nursing-related factors, management and organizational factors, and the creation of a positive work environment. Meanwhile, Fernández et al. [17] analyzed the literature on the political role of nurses over a 10-year period but did not derive the concept of barriers or facilitators to nurses' political participation or policy interventions through qualitative synthesis. In addition, the study by Chiu et al. [18] was not in terms of nurses' social and political activities but rather focused on nursing representative organizations and labor unions. Like this, most of the studies were limited to systematic reviews or scoping reviews rather than deriving concepts by meta-synthesizing individual qualitative studies on barriers or facilitators to nurses' political participation or involvement in health policy.

Therefore, this study aimed to explore the barriers and facilitators to nurses' political participation and healthcare policy intervention through a systematic review and meta-synthesis of previous studies.

## 2. Materials and Methods

### 2.1. Research Design

**Figure 1:**
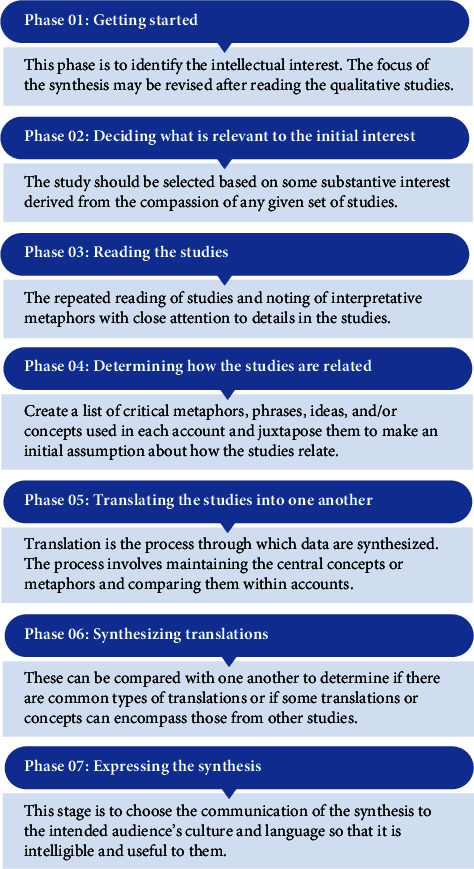
The phases of the meta-ethnography approach by Noblit and Hare (1988).

This study was designed as a qualitative meta-synthesis study that provides a broad understanding of social phenomena by integrating the results of qualitative research [19] to explore the barriers and facilitators influencing nurses' political participation and healthcare policy intervention using meta-ethnography [20]. There are a variety of major qualitative meta-synthesis methods, including meta-ethnography [20], grounded theory synthesis [21], and critical interpretive synthesis [22]. Among these, the meta-ethnography method, which consists of seven stages by Noblit and Hare and is suitable for higher-level analysis and the formation of new interpretations above the discoveries of individual qualitative studies [20], was used for this study (Figure 1). The review question was as follows: what are the barriers and facilitators that influence nurses' political participation or healthcare policy intervention? This study was registered in the PROSPERO International Prospective Register of Systematic Reviews (registration ID: CRD42022346992).

### 2.2. Search Strategy

A systematic literature search was conducted using the COre search electronic databases of COSI (COre Standard, Ideal) presented by the National Library of Medicine (NLM): PubMed, CINAHL, Medline, Embase, Web of Science, and Scopus [19]. A manual search was performed using Google Scholar for a comprehensive literature search. First, two researchers, professors in the Department of Nursing with extensive experience in healthcare policy interventions and qualitative research, independently conducted preliminary searches and established a search strategy while mutually confirming the correspondence between the searched literature. The search strategy was Nurse^∗^ OR Registered nurse^∗^ OR Licensed nurse^∗^ OR Nursing staff OR Nursing personnel OR Nursing profession AND Policy OR Policies OR Politics OR Political OR Advocac^∗^ AND Empirical research OR Focus group^∗^ OR Experience^∗^ OR Qualitative^∗^ OR Interview^∗^ OR Semistructured OR Semistructured OR Unstructured OR In-depth OR In-depth OR Face-to-face OR Grounded theory OR Phenomenolog^∗^ OR Ethnograph^∗^ OR Fieldwork OR Fieldwork (see Supplementary Materials). To select articles, two researchers reviewed the title, abstract, and full text using Endnote 20 after excluding duplicate studies according to the selection or exclusion criteria. In addition, a consensus was reached on inconsistencies in this process after sufficient discussion between the two researchers (Supplementary Table 1).

### 2.3. Study Selection and Quality Assessment

#### 2.3.1. Selection Criteria

Based on the PICOTS-SD criteria, the participants of this study were nurses. Articles that analyzed nursing students, non-nurses, or nurses with other healthcare professionals or politicians were excluded (Table 1). The phenomenon of interest and the main outcomes were to explore the barriers and facilitators that influence nurses' political participation in healthcare policy interventions. The search was conducted from January 20, 2021, to April 28, 2023, and targeted all journals (including doctoral dissertations) in English published before December 31, 2023. Articles on hospital organization or patient advocacy, policy analysis or development, nursing policy education, and practical experience in nursing were excluded. This study design included phenomenology, ethnography, qualitative descriptive studies, and grounded theory. Articles on quantitative research, letters, and editorials were excluded.

#### 2.3.2. The Result of Study Selection

**Figure 2:**
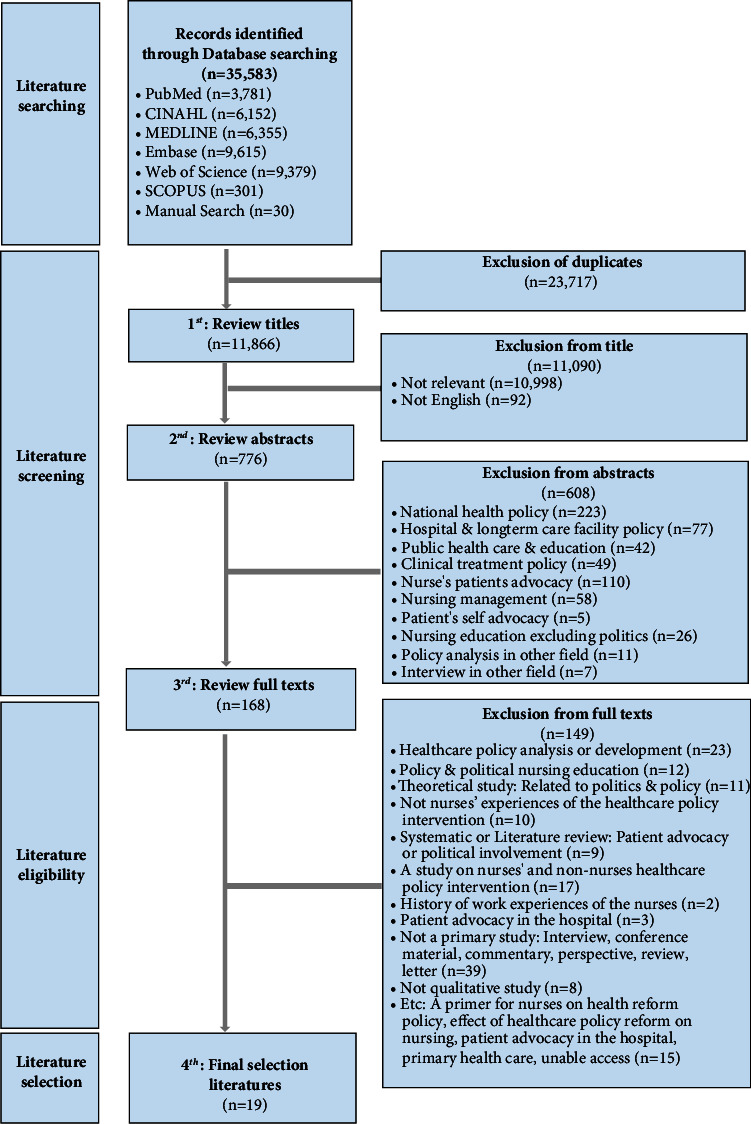
PRISMA (2020) flow diagram of the study selection process.

A total of 35,583 studies were identified, of which 19 individual studies were included in the qualitative meta-analysis using the Preferred Reporting Items for Systematic Reviews and Meta-Analyses (PRISMA) guideline [23]. The PRISMA 2020 guideline provides reporting checklist for systematic reviews by consisting of a 27-item checklist, expanded recommendations, an abstract checklist, and revised flow diagrams to reflect methodological advancements in study identification, selection, appraisal, and meta-synthesis [23]. The databases initially searched for documents were PubMed 3,781, CINAHL 6,152, Medline 6,355, Embase 9,615, Web of Science 9,379, Scopus 301, and Google Scholar 30. After excluding 23,717 duplicate articles, the titles of 11,866 articles were reviewed. After excluding 11,090 articles unrelated to the research, the abstracts of 776 articles were reviewed. The full texts of 168 articles were then reviewed, excluding 608 articles that analyzed national health policy, hospital or long-term care institution policy, policy education, and nursing administration policy. Consequently, 149 articles that were not related to nurses' political or health policy intervention activities were excluded (Figure 2) (Supplementary Table 2). The criteria and processes for selecting studies to be analyzed were carried out in continuous discussion with two researchers. The result of the PRISMA 2020 assessment for reporting systematic reviews is in Supplementary Table 3.

#### 2.3.3. Quality Assessment and Characteristics of the Selected Individual Studies

To evaluate the quality of the final included individual studies, this study used the Critical Appraisal Skills Program (CASP) qualitative checklist, which is commonly used to assess studies and ensure the internal validity of qualitative research meta-synthesis [24]. To reduce the bias of individual studies and secure the internal validity of meta-analyses, evaluating the quality of the primary study to be analyzed is a top priority [25]. The CASP is a tool used to evaluate the reliability, truthfulness, and rigor of qualitative research and consists of ten questions. The higher the score satisfying the questions, the more likely it was that an individual study was systematically conducted. Individual studies were evaluated based on ten questions on the CASP criteria to determine whether they were satisfied. Each researcher independently evaluated and compared the results. In cases of disagreement, consensus was reached through discussion. According to the CASP criteria, 94.7% (*n* = 18) of 19 articles met a high (*n* = 17) [7, 10, 11, 13, 14, 26–37] or high-moderate (*n* = 1) [38] level of research methodological quality (≥0.7), and one article [39] had a low-moderate quality level (0.5). In general, CASP score of less than 0.7 indicates low methodological quality; thus, the meta-analysis results derived from inputting individual studies whose quality has not been verified are challenging to secure internal validity; therefore, one low-quality study was excluded [39] (Table 2).

The 18 studies included in the analysis were conducted in the United States (*n* = 7), Brazil (*n* = 2), Ghana (*n* = 2), Iran (*n* = 2), New Zealand (*n* = 1), Canada (*n* = 1), South Korea (*n* = 1), Thailand (*n* = 1), and Kenya (*n* = 1). Of these, 16 individual articles used qualitative research methods: phenomenology, ethnography, grounded theory, and descriptive analysis, and two articles used mixed methods [32, 35]. For data collection, individual in-depth interviews, focus group interviews, and the Delphi technique were used through purposive sampling. Eleven articles received prior research ethical approval. The participants used in the analysis were 329 nurses, general nurses or nurse managers in hospitals, community public health nurse leaders, civic group activists, government officials, elected politicians, professors, and healthcare policy committee members or policymakers. They had extensive experiences in political activities and healthcare policy reforms in the National Assembly, public institutions, central or local governments, government committees, nursing colleges, nursing representative organizations, communities, civic groups, communities, and hospitals. The research topic explored not only the political and policy reform activities of nurses but also the concepts of barriers and facilitating factors, political competence, advocacy, and strategies based on their experiences (Table 3) (Supplementary Table 4).

### 2.4. Data Extraction and Synthesis

#### 2.4.1. Data Extraction

The researchers independently extracted qualitative data for the study using the format presented by the Joanna Briggs Institute [40]. Any discrepancies between researchers during the extraction process were corrected through joint discussion. Data extraction was performed with reference to the categories or topics specified by the original authors, focusing on research objectives, phenomena of interest, methodology, participant characteristics, and results. ATLAS. ti version 24, qualitative data analysis software, was used to enter and categorize the data to facilitate a more systematic approach to data extraction and analysis. ATLAS. ti is a qualitative data analysis tool. Thus, it can be used to classify, sort, and code input data, which can help researchers explore and understand the nature of the phenomenon of interest. After analyzing the quotes from the individual papers in this study, 232 findings were derived: 114 barriers and 118 facilitators of nurses' political participation or involvement in health policy. The findings were then synthesized into categories of personal, organizational, and professional factors, resulting in eight themes and 22 subthemes for barriers and seven themes and 21 subthemes for facilitators (Table 4) (Supplementary Table 5).

#### 2.4.2. Synthesis by Meta-Ethnography

In this study, a qualitative meta-synthesis analysis was performed based on the meta-ethnography [20] developed by Noblit and Hare in the field of education in the 1980s. This method clarifies the purpose and focus of the research, selects individual studies that fit the research topic, reads individual study results several times, extracts data on the characteristics and analysis of the papers, and identifies common themes and concepts. It determines the relevance between individual study results, further specifies the identified themes and concepts, translates them into each other (first- and second-order constructs), and based on this, synthesizes them into new, more developed concepts (third-order constructs). The interpretative process compares individual research results to explore commonalities and contradictions (reciprocal translation), refutes the relevance of contradictory explanations (refutational synthesis), and uses argument-synthesis strategies to provide new insights. The final step is completed by representing the synthesized results.

The purpose and reason for using meta-ethnography were presented in the first stage of this study. In the second stage, the data were extracted using ATLAS. ti to analyze the selected individual study results after a systematic literature search of the database. In the third stage, after reading the contents of the individual studies several times, the characteristics and results of individual studies were extracted and organized on a data extraction sheet, and notes were used to identify important data. In stage 4, ATLAS. ti was used to identify the relevance and concepts of individual study results, and each author separately coded all sentences by line; if unclear, a mutual discussion process was used to reach an agreement. According to Barnett-Page and Thomas [25], the translation of research participants is classified into “first-order constructs,” the researcher's translation in the primary study forms “second-order constructs,” and the translation provided through qualitative meta-synthesis is classified into “third-order constructs” at various levels of interpretation. Therefore, the researchers analyzed both first- and second-order constructs to explore the barriers to and facilitation of political participation and policy intervention by nurses. Coding was repeated until the themes and concepts of the statements were clarified. Subsequently, researchers classified themes and concepts using tables to enhance their understanding of the research phenomena and analyze the relevance and impact of each concept. In stage 5, the coding results were translated, themes and concepts were further specified to reflect the meaning of each individual study, initial codes and themes were reviewed, and similar themes were combined. As a result of the analysis, the statements of the individual study participants and interpretation authors were similar, and better information on barriers and facilitators was provided. In stage 6, the translated concepts and the relationships between them were used to create new arguments. During this process, the researchers engaged in ongoing discussions while sharing mutual feedback and insights. In the final step, the results of the synthesis of concepts using meta-ethnography are shown in the following tables (Tables 3 and 4), and the reliability of the qualitative evidence synthesis findings was tested using the GRADE-CERQual assessment [41] (Supplementary Table 6). This approach facilitated the assessment of the reliability of the results derived through the synthesis of qualitative research [41]. The authors have diverse academic backgrounds, such as majors in nursing or public health and teaching community health nursing and nursing management, as well as experience in various healthcare policy advisory roles in Korea, concept development, measurement tool development, and qualitative research. Therefore, the authors demonstrated sufficient capabilities for deriving in-depth and insightful research results during each analysis process. The result of the eMERGe reporting criteria for meta-ethnography is in Supplementary Table 7.

## 3. Synthesis Findings

### 3.1. Barriers' Factors

The eight themes were inductively synthesized as follows: personal factors (lack of political interest of nurses and lack of political competence of nurses), organizational factors (nurses' working environment constraints and barriers to organizational culture), and professional factors (stereotypes of the nursing profession, lack of political nursing education, limitation of support for nurses by nursing representative organization, and lack of political power of nursing representative organization).

#### 3.1.1. Personal Factors

Personal barriers affecting nurses' political participation or policy interventions were synthesized into two themes and eight subthemes: the “lack of political interest of nurses” (subthemes: gap in personal beliefs and values and lack of political efficacy) and the “lack of political competence of nurses” (subthemes: deficiency of political knowledge and information, lack of political skill, lack of participation in activities of nursing representative organizations, lack of political participation, and lack of awareness of the policy process).

Most nurse activists who participated in the study stated that the “lack of political interest of nurses” was one of the personal barriers to political participation [7, 11, 13, 14, 26–28, 33, 36, 37]. In particular, it was reported that nurses' interest in political participation varies depending on nurses' gap of political beliefs and values [7, 11, 13, 14, 26–28, 33, 36, 37], and it tends to be low due to the lack of political efficacy of nurses' political participation influencing the field of healthcare policy [7, 10, 11, 14, 26, 28, 33, 36, 37].

They also reported that most nurses' “lack of political competence” [7, 10, 11, 13, 14, 27–29, 33, 36–38] is due to the lack of political knowledge and information [7, 10, 11, 13, 14, 27–29, 33, 36, 37] regarding political processes, policy development, and advocacy strategies, as well as lack of political skills such as communication skills and political networking [7, 10, 11, 13, 14, 28, 29, 33, 36]. In addition, although healthcare policy interventions were feasible to maximize the strengths of individual nurses through collective action [34], they face a limitation that nurses' lack of participation in activities of nursing representative organizations diminishes their collective voice in policy-making [28, 33, 36, 38]. The lack of political participation reflects nurses' minimal involvement in political activities, such as advocacy, lobbying, and interaction with legislators or policymakers [7, 10, 11, 14, 26, 28, 29, 33, 36, 37]. In addition, nurses' lack of awareness of the policy process, without a clear grasp, may make them ill-equipped to contribute to policy discussions or effectively advocate for policy changes [7, 10, 11, 14, 26, 28, 29, 33, 36, 37].

#### 3.1.2. Organizational Factors

The organizational barriers affecting nurses' political participation or policy interventions were inductively synthesized into two themes and five subthemes: “nurses' working environment constraints” (subthemes: poor working environments and time and resource constraints) and “barriers to organizational culture” (subthemes: nepotism and favoritism within the organization, hierarchical and structural organizational culture, and generation differences among nurses).

Poor working environments [10, 11, 26, 28, 31, 33, 36], as a subtheme of “nurses' working environment constraints,” are a significant barrier to nurses' political participation by limiting their capacity and motivation to engage in activities beyond their immediate clinical responsibilities [10, 11, 26, 28, 31, 33, 36]. A challenging work environment characterized by high stress, shift work, long hours, insufficient staffing levels, and a lack of resources leaves nurses physically and emotionally drained, reducing their availability and energy for political advocacy or policy-making activities. Furthermore, poor working environments create time and resource constraints for nurses, which act as a barrier to political participation because it requires time, resources, and sacrifice at an individual level [10, 11, 14, 26, 28, 33, 36].

The subtheme of nepotism and favoritism within the organization under the theme “barriers to organizational culture” refers to practices within healthcare and nursing educational institutions where opportunities for involvement in policy-making are unfairly allocated based on personal relationships rather than merit, skills, or professional qualifications [26, 28, 33, 34, 36]. This creates significant barriers for nurses who seek to participate in political and policy intervention activities but find themselves excluded or marginalized because of these biased practices. Furthermore, the hierarchical and structural organizational culture within healthcare institutions, with rigidly stratified and formal organizational structures, may impede nurses' political participation [10, 26, 28, 30, 31, 33, 36, 37]. Meanwhile, “generational differences among nurses” may act as barriers to cohesive action and participation in policy intervention due to the varying levels of interest, engagement, and methods of communication preferred by different generations [27, 28].

#### 3.1.3. Professional Factors

The professional barriers affecting nurses' political participation or policy interventions were inductively synthesized into four themes and 10 subthemes: “stereotypes of the nursing profession” (subthemes: undervaluation of nurses' expertise and gender biases), “lack of political nursing education” (subthemes: insufficient education and training in political competence and lack of mentorship and legislative internship), “limitation of support for nurses by nursing representative organization” (subthemes: insufficient advocacy efforts for nurses, deficiency of political resources, and minimal encouragement for political participation), and “lack of political power of nursing representative organizations” (subthemes: lack of political network-building and communication skills, lack of conflict management capabilities among interest groups, interaction barriers with legislators and difficulty articulating nursing perspectives, and insufficient intervention in policy decision-making processes).

Relating to the “professional stereotypes of the nursing profession,” the subtheme of undervaluation of nurses' expertise reflects a systemic issue in which nurses' knowledge and contributions are not fully valued in society or the political arena [7, 10, 14, 26, 28–30, 33, 36]. In addition, the nursing profession, historically and predominantly female, faces gender biases that further compound the issue of undervaluation [13, 26, 28, 30, 31, 33, 36, 37]. In a world where men are primarily responsible for policy-making roles, the gender limitations of women in nursing professions also negatively affect performing policy-making functions [13, 26, 28, 30, 31, 33, 36, 37].

Most nurse activists pointed out that the “lack of political nursing education” is a serious professional barriers that undermine nurses' political participation [7, 10, 11, 14, 26–30, 33, 34, 36–38]. In this regard, most nurse activists pointed out insufficient education and training in political competence [7, 10, 11, 14, 26–30, 33, 34, 36–38] and a lack of mentorship and legislative internship [7, 10, 11, 26, 28, 29, 33, 36], especially political mentorship, which is crucial for developing nurses' political skills and policy competence [28, 32, 33, 36]. Without the guidance and support of an experienced mentor, aspiring nurse activists may struggle to find a pathway to political engagement, develop the necessary confidence and skills, and navigate the challenges of policy advocacy and political participation [7, 10, 11, 26, 28, 29, 33, 36].

The subthemes of insufficient advocacy efforts for nurses [7, 28, 33, 34, 36, 37], deficiency of political resources, and minimal encouragement for political participation [10, 28, 29, 33, 36, 37] under the theme “limitation of support for nurses by the nursing representative organizations” were also reported as professional barriers. Nurse activists pointed out that although nursing representative organizations are making efforts to improve the poor working environment of nurses and defend the public's right to health, it is still insufficient, and improvements are needed [10, 28, 29, 33, 36]. In addition, without encouragement and support for political participation from representative organizations, nurses may not perceive political participation as a professional responsibility [34].

Furthermore, most nurse activists stated the “lack of political power of nursing representative organizations [10, 11, 14, 26, 28, 33, 34, 36].” Relating to this, the specific barriers are not only a lack of political network-building and communication skills and conflict management capabilities among interest groups [10, 11, 14, 26, 28, 29, 33, 34, 36] but also interaction barriers with legislators and difficulty articulating nursing perspectives [10, 11, 14, 26, 28, 33, 34, 36] and insufficient intervention in policy decision-making processes [7, 10, 11, 14, 26, 28, 29, 31, 33, 34, 36, 37]. In particular, the lack of political network-building indicates a lack of platforms or environments that facilitate meaningful connections between nurses, policymakers, or influencers in the health policy arena [10, 11, 28, 33, 34, 36]. These limitations restrict the ability of nursing professionals to form alliances, share knowledge, and collaborate on policy initiatives, significantly reducing their influence on health policy interventions [34]. Meanwhile, nurses often encounter competition from other healthcare professionals during policy-making [11]. Therefore, interprofessional competition may manifest as a struggle for influence, recognition, and resources, making it challenging for the nursing profession to assert its interests and contributions [34]. In addition, nurse activists reported feeling excluded from decision-making processes in health policy, which could lead to difficulty in articulating nursing perspectives in policy-making.

### 3.2. Facilitators Factors

The seven themes were inductively synthesized as follows: personal factors (themes: recognition of social responsibilities, enhancing nurses' political competence), organizational factors (themes: innovating organizational environments), and professional factors (themes: enhancing political nursing education, promoting a supportive system by nursing representative organizations, activating nursing organizations' political activities, and enhancing nursing representative organizations' intervention in healthcare policy reform).

#### 3.2.1. Personal Factors

The personal facilitators affecting nurses' political participation or policy interventions were inductively synthesized into two themes and five subthemes: “recognition of social responsibilities” (subthemes: awareness of healthcare problems, enhancing nursing professional values) and the “enhancing nurses' political competence” (subtheme: accumulation of political knowledge, information, and skills, engagement in nursing representative organizations' activities, and participation in political activities or policy intervention).

The motivation for nurse activists' participation in political or healthcare policy interventions was awareness of healthcare problems affecting the public's right to health and nurses' working environments and recognition of social responsibilities to advocate for them as a nursing profession [7, 10, 11, 14, 27–38]. Therefore, increasing nurses' “recognition of social responsibilities” through awareness of healthcare problems [7, 10, 11, 14, 27–38] and enhancing nursing professional values [7, 11, 28, 30–38] are factors in encouraging a commitment to political participation or policy intervention among nurses.

Most nurse activists emphasized “enhancing nurses' political competence” [7, 11, 13, 14, 27–29, 31–36, 38]. They tried strengthening inner political power by accumulating political knowledge, information, and skills on public health problems and policy-making [7, 11, 13, 14, 27–29, 31–36, 38]. They also began to lead healthcare policy reform based on internal and external power accumulated through active political activities, that is, political competence [33, 36]. During this process, they actively participated in political activities within the nursing profession [7, 11, 13, 14, 27–29, 31–36, 38]. Therefore, it is important to enhance political competence and encourage participation in political activities or policy interventions [7, 11, 14, 27, 28, 31–38] through the accumulation of political knowledge, information, and skills [7, 11, 13, 14, 27–29, 31–36, 38] and engagement in nursing representative organization' activities [7, 11, 14, 27–29, 32–36, 38].

#### 3.2.2. Organizational Factors

The organizational factors affecting nurses' political participation or policy interventions were inductively synthesized into one theme and two subthemes: “innovating organizational environments” (subthemes: improvement of nursing work environments and postering supportive organizational culture).

To improve poor working environments, which is one of the organizational barriers, efforts are needed to ensure adequate staffing levels, improve nursing working environments [11, 28, 33, 36], and provide adequate compensation to nurses [27, 28]. Also, by improving the job satisfaction and morale of nurses through “innovating organizational environments,” nurses could be encouraged to participate more actively in political activities [10, 11, 28, 33, 36]. In addition, to overcome the limitations of organizational culture, such as favoritism, hierarchical and structured organizational culture, and generational differences, nursing organizations and healthcare institutions need to foster a culture of collaboration and inclusivity [27, 28]. Furthermore, encouraging open communication, shared decision-making, and flattening hierarchical structures are necessary for postering supportive organizational culture, which could promote active policy debate and political competence of nurses [10, 11, 28, 33, 36].

#### 3.2.3. Professional Factors

The professional factors affecting nurses' political participation or policy interventions were inductively synthesized into four themes and 14 subthemes: “enhancing political nursing education” (subthemes: development and operation of systematic nursing political education curriculum, activating political nursing education for nurturing nursing political activists, development and operation of experiential mentoring and legislative programs, and strengthening evidence-based research for policy development), “promoting a supportive system for nurses by nursing representative organizations” (subthemes: establishing a politically supportive system for nursing activists and enhancing cohesiveness in nursing representative organizations), “activating nursing organizational political activities” (subthemes: building political networking as a source of power, persuasion using effective communication in the political arena, enhancing various political activities through developing networks, and formation of social opinion using media), and “enhancing nursing representative organizations' intervention in healthcare policy reform” (subthemes: development of healthcare policy reform alternatives, lobbying and petitioning policymakers to reflect the nursing perspective, participating in healthcare policy-making process, and implementing and ongoing monitoring of proposed policy reform legislation).

Among the professional factors promoting nurses' political participation, the most frequently stated by nurse activists was “enhancing political nursing education” [7, 11, 14, 26, 28, 29, 32–38], and they reported that education promotes nurses' professional values such as identity, vision, passion, and confidence [11, 28, 32–36, 38]. Most of them stated that they obtained political knowledge, skills, and information through work or political experience more than formal nursing education due to the lack of formal education [11, 13, 26–28, 33–37]. Accordingly, development and operation of systematic nursing political education curriculum, a variety of formal and informal education, are required for nurses [7, 11, 14, 26, 28, 29, 32–38]. In addition, the developing and operating of experiential mentoring and legislative programs are effective in activating political nursing education and nurturing nursing political activists [7, 11, 26–29, 33, 36, 38]. By providing training in political skills for nurses to improve practical interpersonal skills such as communication, persuasion, and negotiation, it could develop nurses' political competence [7, 11, 26–29, 33, 36, 38]. Furthermore, strengthening evidence-based research for policy development is critical for reflecting nursing perspectives into healthcare policies [7, 11, 13, 14, 28, 29, 32–36, 38].

Some nurse activists emphasize “promoting a supportive system by nursing representative organizations” [10, 11, 13, 14, 27, 28, 31–34, 36] through the establishment of a politically supportive system for nursing activists [10, 11, 14, 28, 31, 33, 34, 36]. In addition, to secure political opportunities for nurses, who account for most healthcare professionals, it is crucial to enhance cohesiveness in nursing representative organizations or civic organizations [11, 13, 27, 28, 31, 33–36]. This makes it possible contributing to a unified social voice of the nursing profession [34].

Meanwhile, most nurse activists stated that building political networking as a source of power [7, 11, 13, 14, 27–29, 31–38] is most important for “activating nursing organizations' political activities [7, 11, 13, 14, 27–29, 31–38].” Networking is a strategy that influences health policy and contributes to expanding nurses' political participation by securing a basis for communication and support from politicians, including members of the National Assembly [11, 34]. In addition, establishing social solidarity with various patient groups, healthcare representative organizations, and municipal government organizations is effective in exercising political influence by nursing organizations' enhancing various political activities through developing networks [7, 11, 13, 14, 28, 29, 31, 33–37]. Mainly, it is necessary to be familiar with the language and laws used by politicians and to conduct persuasion using effective communication in the political arena, such as writing, listening, presentation, and conflict resolution skills with perseverance [7, 11, 14, 28, 29, 32–36, 38]. In addition, one of the effective facilitating strategies is the formation of social opinion using media, such as letters, campaigns, parliamentary debates, public hearings, voting, securing political funds or sponsoring politicians, and lobbying [7, 11, 14, 28, 33–37].

Relating to “enhancing nursing representative organization's intervention in healthcare policy reform [7, 11, 13, 14, 27–29, 31–38],” nurse activists sought policy intervention by identifying issues and analyzing healthcare problems, then development of healthcare policy reform alternatives [7, 11, 13, 14, 27–29, 31–36] ,and lobbying and petitioning policymakers to reflect the nursing perspective [7, 11, 13, 14, 28, 29, 33, 34, 36, 37]. Nurse activists emphasized that healthcare policy reform alternatives should be developed first to activate healthcare policy intervention [7, 11, 13, 14, 27–29, 31–36]. Thus, it is proactively necessary to understand issues and policy-making processes to predict current and future healthcare problems [7, 11, 13, 14, 27–29, 31–36]. Then, lobby or petition to persuade legislators, elected officials, and politicians [7, 11, 13, 14, 28, 29, 33, 34, 36, 37] and those experiences create opportunities for participating in the healthcare policy-making process as members of the government policy deliberation committee, members of parliament, and city councilors [7, 10, 11, 14, 27–29, 31–38]. This ultimately contributes to the realization of the proposed healthcare policy reform legislation reflecting the nursing perspective [11, 13, 29, 33, 34, 36]. In this process, nurses must implement and monitor the proposed policy reform legislation to properly implement the reform policies or bills without distortion in the field [11, 34].

## 4. Discussion

This study conducted a systematic review and meta-synthesis to analyze the barriers and factors influencing nurses' political participation and healthcare policy intervention. Three categories, personal, organizational, and professional, affected the barriers and factors of nurses' political participation or policy intervention.

Among the barriers affecting nurses' political participation or policy intervention, personal factors include their lack of political interest and political competence. The organizational barriers were reported as limitations of nurses' work environments and barriers to organizational culture. Furthermore, professional barriers were noted, such as stereotypes of the nursing profession, a lack of political nursing education for nurses, the political power of nursing representative organizations, and the limited support for nurses from these organizations. Relating this, previous studies in political science suggest that political interest is a significant predictor and motivator of political activity [4, 42]. However, nurse activists mentioned that most nurses' political interests were insufficient [6–9, 11, 13–15, 26–28, 33, 36, 37]. In this regard, political scientists Clore and Huntsinger [43] emphasized the importance of creating a public atmosphere because people who lack political knowledge or information generally make vague judgments that negatively affect political participation. Therefore, it is necessary to refer to research results that show a high correlation between political interest in social issues or efficacy and political participation in political science research [44–46]. Meanwhile, nurse activists pointed out the environmental factors of shift work, lack of nursing staff, and gender issues, which are consistent with previous studies suggesting heavy workload, time constraints [10, 11, 26, 28, 33, 36], and male-centered gender issues [13, 26, 28, 33, 36]. Therefore, to revitalize the participation of nurses in politics, improvements in the working environment and sexual problems must precede. Regarding the lack of participation in nursing organizations and activities due to political barriers, most nurses were not members of professional organizations, and the results coincide with those of previous research [7, 47]. Another barrier to nurses' political participation is the lack of nursing education, which has been continuously raised in previous studies [7, 10, 11, 14, 26–30, 33, 34, 36, 38]. According to political scientists, this barrier can be addressed through political socialization, the process of learning, and internalizing the political culture, knowledge, and value attitudes of an individual society [48].

The factors affecting nurses' political participation and policy interventions as personal factors were recognizing social responsibilities and enhancing political competence. In addition, professional factors such as enhancing political nursing education, promoting support systems for nurses by nursing representative organizations, activating nursing organizational and political activities, and enhancing nursing representative organizations' interventions in healthcare policy reforms were noted. Nurse activists began their political activities when they recognized the social responsibility of the nursing profession, which coincided with that presented in previous studies as professional nursing responsibilities [6, 8, 9, 15]. In this regard, Cohen et al. [49] suggested buy-in, self-interest, political sophistication, and leading role as the stages of nursing professionals' political participation. Among them, the perception of political activism was emphasized to achieve the purpose of the nursing profession in the first stage of political participation [49], consistent with the results of this meta-analysis, which recognized healthcare problems and social responsibility as most nurse activists began their activities.

In addition, factors of political participation or policy intervention included promoting awareness of nursing profession values, acquiring political knowledge and information, training political skills by enhancing nursing education, strengthening cohesiveness in professional organizations, building political networking, effective communication, and collaboration, and participating in various political activities to enhance nurses' political competence. First, reinforcing the value for nursing professionals derived from the synthesis of factors promoting political participation refers to nursing identity, confidence, and social advocacy responsibility. Almond and Verba [50], political scholars who first proposed the concept of political competence in political science, related it to personal political competence, political efficacy, objective political competence, and political self-confidence. It may be necessary to prepare measures to improve expectations, attitudes, and confidence in politics that increase political efficacy [50]. More importantly, nurses' pride in the nursing profession must be high to realize their social responsibility and provide opportunities to actively participate in politics actively [9]. Therefore, the development and operation of formal or nonregular curricula, mentoring, and internships at universities or nursing organizations are essential to strengthen attitudes as nursing professionals, political knowledge, and technical education through political socialization.

Meanwhile, nurse political activists not only built political knowledge, information, and political skills such as networking but also actively performed various social solidarity activities and media use [7, 11, 13, 14, 27–29, 31–38]. Like this, strengthening political competence is crucial to activate nurses' political participation and policy intervention. The concept of nurses' political competence, which was derived as a factor that facilitates nurses' political participation, was developed by Han and Kim [9] in 2020 by integrating the concepts presented in previous nursing studies and political science with nurse political activists' interview during the fieldwork phase and categorized into political knowledge, political efficacy, political interaction, and political activity. Previous studies in political science have reported that political competence acts as a motivation or attitude variable and affects the degree of political participation depending on the level of competence [3, 4, 50]. Therefore, since nurses' political participation is possible when the level of political competence is high [9], it is necessary to maximize political influence by improving political competence based on nursing representative organization-centered solidarity [9]. Furthermore, the process through which nurse activists lead policy reform by developing healthcare policy alternatives is consistent with the leading role stage suggested by Cohen et al. [49]. Through these steps, nurses' political competence was strengthened, reflecting the views of the nursing profession when enacting laws or policies on national health and enabling them to advocate for public health rights [42]. Finally, strategies for activating policy intervention and developing policy reform alternatives should be prioritized by conducting healthcare policy analyses and research based on an understanding of healthcare problems and policy decision-making processes. Activation of policy intervention refers to the reinforcement of policy competencies, consistent with Arabi et al. [51], suggesting policy awareness, nurse power, and advocacy of the nursing profession as attributes of the concept of policy impact. This is also consistent with previous studies showing that nurses used academic preparation for policy development for policy interventions, evidence-based research ability to influence policy decision-making based on awareness of health policy issues and understanding of legislative policy processes [26]. Therefore, nurses should activate entry into the political arena by resolving barriers to political participation based on the lessons learned from nurse activists' political experiences and by strengthening their political and policy competencies to advocate for the public and improve the working environment. Through these, the nursing profession can expand its political influence beyond traditional stereotypes.

This study minimized the omission of research results because it analyzed all papers related to the subject of this study without the limitations of language or publication period. Researchers with extensive experience in qualitative research and healthcare policy interventions conducted sufficient discussions throughout the process, from article selection to analysis, in order to ensure the validity and accuracy of the research results. The limitations of this study are the results of a qualitative meta-synthesis that analyzed the experiences of nurses with different personal backgrounds and in some countries with different political and cultural environments; therefore, the study results cannot be generalized. In addition, the risk of selection bias was not entirely excluded by analyzing the participants without considering environmental conditions such as political activity and policy intervention experiences, roles, and workplaces. Some studies included in the analysis did not meet the methodological quality evaluation criteria, and there is a limitation in the analysis based on papers published in a journal rather than receiving and analyzing original data from the authors of the study.

## 5. Conclusions

This study conducted a systematic review and qualitative meta-synthesis of existing research results to comprehensively analyze the barriers and factors influencing nurses' political participation or healthcare policy intervention. The research results synthesized the barriers and factors of political participation or healthcare policy intervention into three categories: personal, organizational, and professional factors, with eight themes and 22 subthemes for barriers, and seven themes and 21 subthemes for facilitators.

The barriers to political participation or policy intervention of nurses were composed of themes, personal factors (lack of political interest of nurses and lack of political competence of nurses), organizational factors (themes: limitations of the work environments of nurses and barriers to organizational culture), and professional factors (themes: stereotypes of the nursing profession, lack of political nursing education, limitation of support for nurses by nursing representative organizations, and lack of political power of nursing representative organizations). Factors of political participation were composed of themes, personal factors (themes: recognition of social responsibilities and enhancing political competence), organizational factors (themes: innovating organizational environments), professional factors (themes: enhancing political nursing education, promoting a supportive system by nursing representative organizations, activating nursing organizational political activities, and enhancing nursing representative organizations' intervention in healthcare policy reform).

This study can be used as main data for enhancing nurses' political participation, policy interventions, and preparation strategies. The study is significant in where most of the participants in this study have experienced as nurse political activists; there is no previous study on the experience of general nurses' political participation to develop a more detailed strategy for activating political participation in the future. There are some studies on the effects of education, despite the lack of nursing education. However, research on the development of nursing programs is necessary because there are few studies on the development of nursing education. Furthermore, development studies measuring nurses' political competence and theories of strengthening nurses' political competence are needed [45].

## 6. Implications for Nursing Management

Nurses' political participation is required to optimize the quality and equity of health services provided to patients and to improve their working environments [12, 30]. Based on the growth process as a nursing political activist synthesized in this study, the barriers to and facilitating factors for nurses' political participation, and the concept of activating policy interventions, a theory for strengthening nurses' political competencies can be developed. To activate nurses' political participation, nursing organization leaders in practice and in various nursing fields must refer to this study. Through this, nurses can improve their working environment, increase their interest in politics, share political information and knowledge, join the nursing representative organization, and encourage participation in various political activities. Furthermore, instructors in nursing education should organize curricula, develop programs, and systematically implement them to strengthen political nursing education. Finally, based on the results of this study, nursing representative organizations can prepare a strategy for activating nurses' political participation, provide them with opportunities to engage in political activities and policy interventions, and promote the revitalization of politicians.

## Figures and Tables

**Table 1:** Selection criteria for the study.

Classification	Inclusion criteria	Exclusion criteria
Participants	(i) Nurses (ii) Registered nurses (iii) Licensed nurses (iv) Nursing profession	(i) Non-nurse occupations, healthcare personnel, healthcare profession, and other occupation (ii) Nurses with other healthcare professionals or politicians
Interventions/exposure	(i) Nurses' experiences in healthcare, political advocacy, policy intervention, policy reform, or political activities	(i) Non-nurses' experience in political participation or policy reform activities (ii) National/local government policy or political activities (iii) International policy or politics
Comparisons/control	(i) Not applicable	(i) Not applicable
Outcomes: phenomenon of interest	(i) To explore the perception of nurses about their experiences of political participation and healthcare policy reform (ii) To evaluate barriers and facilitators of political participation and healthcare policy intervention	(i) Results of healthcare policy analysis (ii) Policy and political nursing education (iii) Factors of political involvement and political activity of nurses (iv) Effectiveness of healthcare policy reform (v) History of work experiences of the nurses (vi) Patient advocacy in the hospital
Time	(i) Articles published from the first publication to December 31, 2023, by each database of search	(i) Articles published after December 31, 2023
Setting	(i) National/local healthcare policy or advocacy or politics	(i) Organizational policy or politics (ii) Non-English papers
Study design	(i) Qualitative study	(i) Quantitative studies (ii) Mixed methods' research with absent or uncertain qualitative data (iii) Not a primary study: interview, conference material, commentary, perspective, review, and letter
	(ii) Descriptive study	
	(iii) Exploratory study	
	(iv) Empirical research	
	(v) Grounded theory	
	(vi) Phenomenology	
	(vii) Ethnography	
	(viii) Case study	
	(ix) Focus group interviews	
	(x) In-depth interviews	
	(xi) Semistructured interviews	
	(xii) Unstructured interviews	
	(xiii) Face-to-face interviews	

**Table 2:** Results of the appraisal of methodological quality by the CASP criteria.

Studies	1	2	3	4	5	6	7	8	9	10	Total	Score	Quality
Acheampong et al. [26]	1	1	1	1	1	1	1	1	1	1	10	1.0	High
Barry [27]	1	1	1	1	1	1	0	1	1	1	9	0.9	High
Declercq [39]	1	1	0	1	0	0	0	0	1	1	5	0.5	Low-moderate
Deschaine and Schaffer [13]	1	1	1	1	1	1	0	1	1	1	9	0.9	High
Digaudio [28]	1	1	1	1	1	1	1	1	1	1	9	0.9	High
Dollinger [14]	1	1	1	1	1	1	1	1	1	1	10	1.0	High
Donovan et al. [29]	1	1	1	1	1	1	1	1	1	1	10	1.0	High
Hajizadeh et al. [10]	1	1	1	1	1	1	1	1	1	1	10	1.0	High
Hajizadeh et al. [7]	1	1	1	1	1	1	1	1	1	1	10	1.0	High
Han [11]	1	1	1	1	1	1	1	1	1	1	10	1.0	High
Laari and Duma [30]	1	1	1	1	1	1	1	1	1	1	10	1.0	High
Melo et al. [38]	1	1	1	1	1	0	1	0	0	1	7	0.7	High-moderate
Rabelo and Silva [31]	1	1	1	1	1	1	1	1	1	1	10	1.0	High
Shariff [32]	1	1	1	1	1	1	1	1	1	1	10	1.0	High
Taylor [33]	1	1	1	1	1	1	1	1	1	1	10	1.0	High
Warner [34]	1	1	1	1	1	1	1	1	1	1	10	1.0	High
Wichaikhum et al. [35]	1	1	1	1	1	1	1	1	1	1	10	1.0	High
Williams [36]	1	1	1	1	1	1	1	1	1	1	10	1.0	High
Wilson et al. [37]	1	1	1	1	1	1	1	1	1	1	10	1.0	High
Total (%)	100	100	94.7	100	94.7	89.5	84.2	89.5	94.7	100			

*Note.* Score: <0.50: low quality; 0.51–0.65: low-moderate quality; 0.66–0.79: high-moderate quality; ≥0.80: high quality. Q1: was there a clear statement of the aims of the research? Q2: is a qualitative methodology appropriate? Q3: was the research design appropriate to address the aims of the research? Q4: was the recruitment strategy appropriate to the aims of the research? Q5: was the data collected in a way that addressed the research issue? Q6: has the relationship between the researcher and participants been adequately considered? Q7: have ethical issues been taken into consideration? Q8: was the data analysis sufficiently rigorous? Q9: is there a clear statement of findings? Q10: how valuable is the research?

**Table 3:** Characteristics of selected articles.

Study/country	Phenomena of interest	Design/analysis	Setting/context/culture	Participants	Data collection
Acheampong et al. [26], Ghana	Exploring the participation of nurses in healthcare policy development and reforms	Exploratory: descriptive qualitative study-content analysis	Six regions: hospitals, educational institutions, health directorate offices, and the nursing council	15 general nurses and 15 midwives in key leadership positions in their workplaces	Purposive sampling; semistructured individual interviews; open-ended in-depth interviews
Barry [27], the United States of America	Describing the political socialization processes of nurses who had attained specialized roles in the governments	Exploratory: descriptive qualitative study-analysis of responses	Positioning in congressional offices, regulatory agencies, or state legislatures, and willingness to participate	33 nurses who had attained specialized roles in public policy development in the federal and state governments	Purposive sampling; semistructured/open-ended in-depth individual interviews; face-to-face and by telephone
Deschaine and Schaffer [13], the United States of America	Identifying factors that affect the ability of public health nurse leaders to influence health policy	Exploratory: descriptive qualitative study—Longest's (2002) public policy-making framework	A city or county public health agency	Eight PHN leaders representing rural, suburban, and urban in public health nursing	Semistructured individual interviews based on Longest's (2002) public policy-making framework
Digaudio [28], the United States of America	Investigating phenomena of policy-making by nurses as experienced by those in the field	Grounded theory	At least a baccalaureate degree in nursing	20 nurses who could identify and discuss a policy-making activity they experienced	Purposive sampling; semistructured individual interviews; open-ended in-depth interviews
Dollinger [14], the United States of America	Exploring how effectively nurses function as advocates in the federal health policy process	Grounded theory	Current or past experience in the legislature or an executive branch agency of the federal government	11 registered nurses who had experience as staff in government offices, committees, or federal agencies	Purposive snowball sampling; semistructured individual interviews; open-ended in-depth interviews
Donovan et al. [29], New Zealand	Examining perceptions of policy and political leadership in nursing	Exploratory: descriptive qualitative study-constant comparative analysis	Fellows of the college of nurses who volunteered through a standard solicitation of the college, Aotearoa (NZ) Inc.	18 nurse leaders from across the country	Volunteer sampling; semistructured interviews; face to face (16 nurses), telephone (2 nurses)
Hajizadeh et al. [10], Iran	Exploring the barriers and facilitators concerning nurse managers' participation in the health policy‐making process	Exploratory: descriptive qualitative study-thematic analysis	Iranian nationality; nurses ≥25 years; a university degree; worked in administrative positions with an experience of at least 3 years in hospital	16 nurse managers who are involved based on their leadership positions and responsibilities in the hospital or governmental offices	Purposive sampling; semistructured individual interviews; open-ended in-depth interviews
Hajizadeh et al. [7], Iran	Exploring the nurse managers' attitudes and perceived benefits in the health policy-making process	Thematic analysis	Tabriz University of Medical Sciences which has over 15 teaching hospitals northwest of Iran	16 nurse managers, government officials, and faculty members	Purposive sampling; semistructured individual interviews; open-ended in-depth interviews
Han [11], South Korea	Exploring the healthcare policy reform activities of Korean nurses engaged in civic organizations	Phenomenology	Korean civic organizations' activities for more than 5 years with a bachelor's or higher degree	Seven Korean civic activist nurses who had led successful healthcare policy reforms through policy interventions	Purposive; snowball sampling; semistructured individual interviews; in-depth interviews
Laari and Duma [30], Ghana	Exploring situations that thwart nurses from performing their health advocacy role	A qualitative inductive descriptive design by Creswell and Poth; qualitative content analysis by Graneheim and Lundman	Three Ghanaian regional hospitals that served as referral, research, and teaching facilities for nurses	24 professional nurses who met the inclusion criteria in each of the regional hospitals	Purposive sampling; semistructured individual interviews; in-depth interviews
Melo et al. [38], Brazil	Identifying the conception of political participation of the nurse manager in the Brazilian public healthcare system	Exploratory: descriptive qualitative study thematic analysis	Qualified in full municipal management for at least two years and having party-political continuity	Nine manager or comanager nurses who have a position as a PHN with a leadership capacity	Semistructured individual interviews; face to face
Rabelo and Silva [31], Brazil	Identifying expression of sociopolitical knowledge, based on nurses in social movements	Foucault's framework and descriptive analysis	Social movements and women's collectives in the Metropolitan Region of Belo Horizonte, MG, Brazil	Six nurses who have experience in social movements and political representation	Nonprobabilistic and intentional sampling; semistructured individual interviews; in-depth interviews
Jivraj Shariff [32], Kenya	Exploring the extent of nurse leaders' participation and leadership attributes necessary for nurse leaders in health policy development in East Africa	Mixed method: Qualitative, descriptive, and quantitative research; qualitative analysis; statistical analysis	Three East African countries of Kenya, Uganda, and Tanzania	78 national nurse leaders who have experience in health policy development and work at the Ministry of Health, Nursing Councils, etc	Purposive sampling; 3-round Delphi method: round 1: open-ended survey questions; rounds 2 and 3: questionnaires and qualitative/quantitative data
Taylor [33], the United States of America	Eliciting insight from the public policy leaders about their current advocacy initiatives that motivate nurses	Descriptive web-based survey design; Bandura's Social Cognitive Theory (1986); inductive content analysis	Two regional professional nursing organizations were designated as expert mentors	12 executive leadership and board committee members from their respective organizations	Purposive convenience sampling; initial web-based electronic survey; semistructured web interview focuses group sessions
Warner [34], the United States of America	Identifying the skills of political competence in the stories of six politically expert nurse activists	Phenomenology	Having experience in appointed and elected office, organizational leadership, and federal healthcare reform activities	Six politically expert nurse activists	Purposive sampling; semistructured individual interviews; open-ended in-depth interviews
Wichaikhum et al. [35], Thailand	Developing a strategic model of participation in policy development for nurses in Thailand	Mixed method: qualitative, descriptive, and quantitative study—qualitative analysis; quantitative analysis; validity	Having participated in policy development at the organizational or national level and/or working for nursing professional organizations with at least 10-year experience	15 nurse experts who have participated in policy development	Purposive sampling; 3-round Delphi method: round 1—open-ended individual interviews; round 2: assessing the probability of statements; round 3: finding agreement among the panel experts
Williams [36], the United States of America	Gaining understanding of the concept and practice of advocacy aimed at public policy development	Grounded theory	Working in the states in various nursing/political advocacy roles	10 nurses who were active in political advocacy as part of their nursing practice	Purposive sampling; semistructured individual interviews; open-ended in-depth interviews
Wilson et al. [37], Canada	Exploring why and how nurses became politically active and what they achieved	Grounded theory	Canadian nursing organizations to gain volunteers	10 elected or politically active Canadian nurses who had been elected to a political position	Volunteer sampling; semistructured individual interviews; telephone interviews

**Table 4:** Synthesized findings.

Categories	Themes	Subthemes	References	Findings
*Barriers of political participation or policy intervention*				
Personal factors	Lack of political interest of nurses	Gap in political beliefs and values	[7, 11, 13, 14, 26–28, 33, 36, 37]	6
		Lack of political efficacy	[7, 10, 11, 14, 26, 28, 33, 36, 37]	8
	Lack of political competence of nurses	Lack of political knowledge and information	[7, 10, 11, 13, 14, 27–29, 33, 36, 37]	2
		Lack of political skill	[7, 10, 11, 13, 14, 28, 29, 33, 36]	4
		Lack of participation in activities of nursing representative organizations	[28, 33, 36, 38]	3
		Lack of political participation	[7, 10, 11, 14, 26, 28, 29, 33, 36, 37]	2
		Lack of awareness of the policy process	[7, 10, 11, 14, 26, 28, 29, 33, 36, 37]	4
Organizational factors	Nurses' working environment constraints	Poor working environments	[10, 11, 26, 28, 31, 33, 36]	6
		Time and resource constraints	[10, 11, 14, 26, 28, 33, 36]	3
	Barriers to organizational culture	Nepotism and favoritism within the organization	[26, 28, 33, 34, 36]	2
		Hierarchical and structural organizational culture	[10, 26, 28, 30, 31, 33, 36, 37]	7
		Generation differences among nurses	[27, 28]	2
Professional factors	Stereotypes of the nursing profession	Undervaluation of nurses' expertise	[7, 10, 14, 26, 28–30, 33, 36]	5
		Gender biases	[13, 26, 28, 30, 31, 33, 36, 37]	5
	Lack of political nursing education	Insufficient education and training in political competence	[7, 10, 11, 14, 26–30, 33, 34, 36–38]	16
		Lack of mentorship and legislative internship	[7, 10, 11, 26, 28, 29, 33, 36]	7
	Limitation of support for nurses by nursing representative organizations	Insufficient advocacy efforts for nurses	[7, 28, 33, 34, 36, 37]	3
		Deficiency of political resources and minimal encouragement for political participation	[10, 28, 29, 33, 36, 37]	5
	Lack of political power of nursing representative organizations	Lack of political network-building and communication skills	[10, 11, 28, 33, 34, 36]	5
		Lack of conflict management capabilities among interest groups	[10, 11, 14, 26, 28, 29, 33, 34, 36]	5
		Interaction barriers with legislators and difficulty articulating nursing perspectives	[10, 11, 14, 26, 28, 33, 34, 36]	3
		Insufficient intervention in policy decision-making processes	[7, 10, 11, 14, 26, 28, 29, 31, 33, 34, 36, 37]	11

*Facilitators of political participation or policy intervention*				
Personal factors	Recognition of social responsibilities	Enhancing nursing professional values	[7, 11, 28, 30–38]	5
		Awareness of healthcare problems	[7, 10, 11, 14, 27–38]	9
	Enhancing nurses' political competence	Accumulating of political knowledge, information, and skills	[7, 11, 13, 14, 27–29, 31–36, 38]	3
		Engagement in nursing representative organizations' activities	[7, 11, 14, 27–29, 32–36, 38]	5
		Participation in political activities or policy intervention	[7, 11, 14, 27, 28, 31–38]	9
Organizational factors	Innovating organizational environments	Improvement of nursing work environments	[11, 28, 33, 36]	3
		Postering supportive organizational culture	[10, 11, 28, 33, 36]	4
Professional factors	Enhancing political nursing education	Development and operation of systematic nursing political education curriculum	[7, 11, 14, 26, 28, 29, 32–38]	8
		Activating political nursing education for nurturing nursing political activists	[7, 11, 26–29, 33, 36, 38]	6
		Development and operation of experiential mentoring and legislative programs	[7, 11, 27–29, 33, 35–37]	5
		Strengthening evidence-based research for policy development	[7, 11, 13, 14, 28, 29, 32–36, 38]	4
	Promoting a supportive system by nursing representative organizations	Establishing a politically supportive system for nurse activists	[10, 11, 14, 28, 31, 33, 34, 36]	12
		Enhancing cohesiveness in nursing representative organizations	[11, 13, 27, 28, 31, 33–36]	3
	Activating nursing organizations' political activities	Building political networking as a source of power	[7, 11, 13, 14, 27–29, 31–38]	8
		Persuasion using effective communication in the political arena	[7, 11, 14, 28, 29, 32–36, 38]	6
		Enhancing various political activities through developing networks	[7, 11, 13, 14, 28, 29, 31, 33–37]	4
		Formation of social opinion using media	[7, 11, 14, 28, 33–37]	9
	Enhancing nursing representative organizations' intervention in healthcare policy reform	Development of healthcare policy reform alternatives	[7, 11, 13, 14, 27–29, 31–36]	3
		Lobbying and petitioning policymakers to reflect the nursing perspective	[7, 11, 13, 14, 28, 29, 33, 34, 36, 37]	2
		Participating in healthcare policy-making process	[7, 10, 11, 14, 27–29, 31–38]	8
		Implementing and ongoing monitoring of proposed policy reform legislation	[11, 34]	2

## Data Availability

The data used to support the findings of this study are available from the first author upon resonable request.

## Supplementary Materials

Supplementary Materials Supplementary Materials include seven files that provide further information about search strategies, excluded articles based on the full-text review, the PRISMA 2020 checklist, a list of the selected articles for analysis, the findings (barriers and facilitators), the GRADE CERQual assessment, and the eMERGEe reporting result.
