# Supplementary material for: The Barriers and Facilitators Influencing Nurses' Political Participation or Healthcare Policy Intervention: A Systematic Review and Qualitative Meta-Synthesis
Source: J Nurs Manag. 2024 Jun 28;2024:2606855. doi: 10.1155/2024/2606855 (PMC11919103; doi:10.1155/2024/2606855)
Supplement: Supplementary Materials — include seven files that provide further information about search strategies, excluded articles based on the full-text review, the PRISMA 2020 checklist, a list of the selected articles for analysis, the findings (barriers and facilitators), the GRADE CERQual assessment, and the eMERGEe reporting result. [file 2606855.f1.zip › 1_Search strategies.docx]

**Supplementary table 1.** **Database search strategies**

The search strategy was Nurse* OR Registered nurse* OR Licensed nurse* OR Nursing staff OR Nursing personnel OR Nursing profession AND Policy OR Policies OR Politics OR Political OR Advocac* AND Empirical research OR Focus group* OR Experience* OR Qualitative* OR Interview* OR Semi-structured OR Semi-structured OR Unstructured OR In-depth OR In-depth OR Face-to-face OR Grounded theory OR Phenomenolog* OR Ethnograph* OR Fieldwork OR Fieldwork.

| **1. PubMed (1908 – 31 December, 2023)** | | |
| --- | --- | --- |
| #1 | “Nurse*”[mesh] OR “Registered nurse*”[tiab] OR “Licensed nurse*”[tiab] OR “Nursing staff”[tiab] OR “Nursing personnel”[tiab] OR “Nursing profession”[tiab] | 194,087 |
| #2 | “Policy”[tiab] OR “Policies”[tiab] OR “Politics”[tiab] OR “Political”[tiab] OR “Advocac*”[tiab] | 441,888 |
| #3 | “Empirical research”[tiab] OR “Focus group*”[tiab] OR “Experience*”[tiab] OR “Qualitative*”[tiab] OR “Interview*”[tiab] OR “Semi-structured”[tiab] OR “Semistructured”[tiab] OR “Unstructured”[tiab] OR “In-depth”[tiab] OR “Indepth”[tiab] OR “Face-to-face”[tiab] OR “Grounded theory”[tiab] OR “Phenomenolog*”[tiab] OR “Ethnograph*”[tiab] OR “Fieldwork”[tiab] OR “Field work”[tiab] | 2,130,358 |
| #4 | #1 AND #2 AND #3 | **3,781** |
| **2. EBSCOhost CINAHL (1937 – 31 December, 2023)** | | |
| S1 | TI “Nurse*” OR AB “Nurse*” OR TI “Registered nurse*” OR AB “Registered nurse*” OR TI “Licensed nurse*” OR AB “Licensed nurse*” OR TI “Nursing staff” OR AB “Nursing staff” OR TI “Nursing personnel” OR AB “Nursing personnel” OR TI “Nursing profession” OR AB “Nursing profession” | 394,204 |
| S2 | TI “Policy” OR AB “Policy” OR TI “Policies” OR AB “Policies” OR TI “Politics” OR AB “Politics” OR TI “Political” OR AB “Political” OR TI “Advocac*” OR AB “Advocac*” | 199,806 |
| S3 | TI “Empirical research” OR AB “Empirical research” OR TI “Focus group*”OR AB “Focus group*” OR TI “Experience*” OR AB “Experience*” OR TI “Semi-structured” OR AB “Semi-structured” OR TI “Semistructured” OR AB “Semistructured” OR TI “Unstructured” OR AB “Unstructured” OR TI “In-depth” OR AB “In-depth” OR TI “Indepth” OR AB “Indepth” OR TI “Face-to-face” OR AB “Face-to-face” OR TI “Grounded theory” OR AB “Grounded theory” OR TI “Phenomenolog*” OR AB “Phenomenolog*” OR TI “Ethnograph*” OR AB “Ethnograph*” OR TI “Field work” OR AB “Field work” | 614,177 |
| S4 | S1 AND S2 AND S3 | **6,152** |
| **3. EBSCOhost Medline (1946 – 31 December, 2023)** | | |
| S1 | TI “Nurse*” OR AB “Nurse*” OR TI “Registered nurse*” OR AB “Registered nurse*” OR TI “Licensed nurse*” OR AB “Licensed nurse*” OR TI “Nursing staff” OR AB “Nursing staff” OR TI “Nursing personnel” OR AB “Nursing personnel” OR TI “Nursing profession” OR AB “Nursing profession” | 337,602 |
| S2 | TI “Policy” OR AB “Policy” OR TI “Policies” OR AB “Policies” OR TI “Politics” OR AB “Politics” OR TI “Political” OR AB “Political” OR TI “Advocac*” OR AB “Advocac*” | 421,373 |
| S3 | TI “Empirical research” OR AB “Empirical research” OR TI “Focus group*”OR AB “Focus group*” OR TI “Experience*” OR AB “Experience*” OR TI “Semi-structured” OR AB “Semi-structured” OR TI “Semistructured” OR AB “Semistructured” OR TI “Unstructured” OR AB “Unstructured” OR TI “In-depth” OR AB “In-depth” OR TI “Indepth” OR AB “Indepth” OR TI “Face-to-face” OR AB “Face-to-face” OR TI “Grounded theory” OR AB “Grounded theory” OR TI “Phenomenolog*” OR AB “Phenomenolog*” OR TI “Ethnograph*” OR AB “Ethnograph*” OR TI “Field work” OR AB “Field work” | 1,653,927 |
| S4 | S1 AND S2 AND S3 | **6,355** |
| **4. Embase (1966 – 31 December, 2023)** | | |
| #1 | “Nurse*”:ti,ab OR “Registered nurse*”:ti,ab OR ‘Licensed nurse*’:ti,ab OR ‘Nursing staff’:ti,ab OR ‘Nursing personnel’:ti,ab OR ‘Nursing profession’:ti,ab | 421,033 |
| #2 | Policy’:ti,ab OR ‘Policies’:ti,ab OR ‘Politics’:ti,ab OR ‘Political’:ti,ab OR ‘advocac*’:ti,ab | 491,441 |
| #3 | Empirical research’:ti,ab OR ‘Focus group*’:ti,ab OR ‘Experience*’:ti,ab OR ‘Qualitative*’:ti,ab OR ‘Interview*’:ti,ab OR ‘Semi-structured’:ti,ab OR ‘Semistructured’:ti,ab OR ‘Unstructured’:ti,ab OR ‘In-depth’:ti,ab OR ‘Indepth’:ti,ab OR ‘Face-to-face’:ti,ab OR ‘Grounded theory’:ti,ab OR ‘Phenomenolog*’:ti,ab OR ‘Ethnograph*’:ti,ab OR ‘Fieldwork’:ti,ab OR ‘Field work’:ti,ab | 2,843,491 |
| #4 | #1 AND #2 AND #3 | **9,615** |
| **5. Web of Science (1950 – 31 December, 2023)** | | |
| #1 | TS=(‘Nurse*’ OR ‘Registered nurse*’ OR ‘Licensed nurse*’ OR ‘Nursing staff’ OR ‘Nursing personnel’ OR ‘Nursing profession’) | 274,131 |
| #2 | TS=(‘Policy’ OR ‘Policies’ OR ‘Politics’ OR ‘Political’ OR ‘advocac*’) | 1,690,246 |
| #3 | TS=(‘empirical research’ OR ’focus group*’ OR’experience*’ OR ’qualitative*’ OR ’interview*’ OR’semi-structured’ OR ’semistructured’ OR ’unstructured’ OR’in-depth’ OR ’indepth’ OR ’face-to-face’ OR’grounded theory’ OR ’phenomenolog*’ OR ’ethnograph*’ OR’fieldwork’ OR ’field work’) | 4,760,020 |
| #4 | #1 AND #2 AND #3 | **9,379** |
| **6. SCOPUS (1981 – 31 December, 2023)** | | |
| S1 | TI “Nurse*” OR AB “Nurse*” OR TI “Registered nurse*” OR AB “Registered nurse*” OR TI “Licensed nurse*” OR AB “Licensed nurse*” OR TI “Nursing staff” OR AB “Nursing staff” OR TI “Nursing personnel” OR AB “Nursing personnel” OR TI “Nursing profession” OR AB “Nursing profession” | 56 |
| S2 | TI “Policy” OR AB “Policy” OR TI “Policies” OR AB “Policies” OR TI “Politics” OR AB “Politics” OR TI “Political” OR AB “Political” OR TI “Advocac*” OR AB “Advocac*” | 2,372,480 |
| S3 | TI “Empirical research” OR AB “Empirical research” OR TI “Focus group*”OR AB “Focus group*” OR TI “Experience*” OR AB “Experience*” OR TI “Semi-structured” OR AB “Semi-structured” OR TI “Semistructured” OR AB “Semistructured” OR TI “Unstructured” OR AB “Unstructured” OR TI “In-depth” OR AB “In-depth” OR TI “Indepth” OR AB “Indepth” OR TI “Face-to-face” OR AB “Face-to-face” OR TI “Grounded theory” OR AB “Grounded theory” OR TI “Phenomenolog*” OR AB “Phenomenolog*” OR TI “Ethnograph*” OR AB “Ethnograph*” OR TI “Field work” OR AB “Field work” | 1,469 |
| S4 | 1 AND 2 AND 3 | **301** |
