# Supplementary material for: The Barriers and Facilitators Influencing Nurses' Political Participation or Healthcare Policy Intervention: A Systematic Review and Qualitative Meta-Synthesis
Source: J Nurs Manag. 2024 Jun 28;2024:2606855. doi: 10.1155/2024/2606855 (PMC11919103; doi:10.1155/2024/2606855)
Supplement: Supplementary Materials — include seven files that provide further information about search strategies, excluded articles based on the full-text review, the PRISMA 2020 checklist, a list of the selected articles for analysis, the findings (barriers and facilitators), the GRADE CERQual assessment, and the eMERGEe reporting result. [file 2606855.f1.zip › 2_EXCL~1.DOC]

**Supplementary table 2. Excluded Articles Based on Full-Text Review (*n* =149)**

The full texts of 168 articles were then reviewed, excluding 608 articles that analyzed national health policy, hospital or long-term care institution policy, policy education, and nursing administration policy. Consequently, 149 articles that were not related to nurses’ political or health policy intervention activities were excluded.

| **No** | **Author, Year, Country** | **Title of article** | **Type of Publication, Journal** | **Methodology** | **Research objective** | **Exclusion reason(s)** |
| --- | --- | --- | --- | --- | --- | --- |
| 1 | Abbasinia et al.; 2020; Iran | Patient advocacy in nursing: A concept analysis | Research article; Nursing Ethics | Exploratory- Qualitative study; Evolutionary concept analysis | Definition of patient advocacy | Literature review: Related to patient advocacy |
| 2 | Adams;2015; The United States of America | The Influence of Emerging Nursing Strategy and Policy Leaders: An Interview With Dr. Suzanne Miyamoto | Journal interview; The Journal of Nursing Administration | Descriptive interview | Explaining the politics of nursing | Not a primary study: Interview |
| 3 | Adams;2017; United States of America | Influencing Scope of Practice Policy and Health Literacy: An Interview with Dr. Joy Deupree | Journal interview; J Nurs Adm | Descriptive interview | Demonstrating leadership in patient care, practice policy, and research | Not a primary study: Interview |
| 4 | Almeidaet al.; 2020; Brazil | The systems and signs of political militants in/of Brazilian nursing | Research article; Rev Bras Enferm | Historical and qualitative study | Analyzing the systems and signs in the constitution of militant nurses | Not nurses’ experiences of the healthcare policy intervention |
| 5 | Al‐Riyami et al.;2015; Oman | Nurses’ perceptions of the challenges related to the Omanization policy | Research article;International Nursing Review | Exploratory- Descriptive Qualitative study | Exploring the nurses’ perceptions of Omanization policy | Not nurses’ experiences of the healthcare policy intervention |
| 6 | Alvarengaet al.;2012; Brazil | Men’s Health Policy: perspectives of nurses for its implementation | Research article;Revista Brasileira de Enfermagem | Descriptive Qualitative interview | Analyzing the perception of nurses from the Family Health strategy policy | Healthcare policy analysis |
| 7 | Alzaheret al.;2021; Saudi Arabia | Proposing a health audit model for evaluating the health policy to confront the covid-19 pandemic for nursing professionals in Saudi hospitals | Research article; Journal of Cardiovascular Disease Research | Exploratory- Descriptive Qualitative study; Policy analysis | Discovering the role of health audit in evaluating the health policy | Healthcare policy analysis |
| 8 | Akin & Kursun;2020; Turkey | Perception and opinion of nursing faculties regarding advocacy role: A qualitative research | Research article;Nursing Forum | Exploratory- Descriptive Qualitative study | Determining nurse academics’ perception and opinions about advocacy | Theoretical study: Related to patient advocacy |
| 9 | Anders;2020; United States of America | How a lesson in patient advocacy changed my career | Research article;Lippincott Williams & Wilkins | Narrative qualitativestudy | Exploring the nurse’s caring experience for a patient | Patient advocacy in the hospital |
| 10 | Ashton;1989; United States of America | A policy analysis: federal support for nursing research | Thesis (Doctorate); University of Utah | Descriptive Qualitative study; Grounded theory | Analyzing public policy processes related to federal support | Healthcare policy analysis |
| 11 | Batalhaet al.;2016; Brazil | Knowledge of nurses on health policies for the elderly person | Research article; Revista de Enfermagem UFPE | Exploratory- Descriptive Qualitative study | Describing the knowledge of nurses for the elderly policies | Healthcare policy analysis |
| 12 | Beck et al.;2003; Brazil | Participation in the design of a political pedagogical project in a nursing | Conference proceeding; Revista brasileira de enfermagem | Conference material | Tracing the route of the academic community pursuing a Nursing Degree | Not a primary study: Conference material |
| 13 | Bentonet al.;2016; United States of America | An integrative review of pursing policy and political competence | Research article; International Nursing Review | Integrative literature review | Conducting an integrative review identifying the nursing profession’s policy involvement | Literature review of factors the Political Involvement |
| 14 | Bergstrom;1992; United States of America | Nursing war: a philosophical study of the relationship between the profession of nursing and political violence | Thesis (Doctorate); University of Colorado Health Sciences Center | Exploratory- Descriptive Qualitative study; Concept analysis | Exploration nature of nursing’s interactions with war | Theoretical study: Related to patient advocacy |
| 15 | Beliera;2019; Argentina | The professionalization of nursing in Neuquen. Process of construction of a labor and political collective | Research article; Trabajos Y Comunicaciones | Mixed-method study design | Analyzing the professionalization of nursing and the process of building a labor | Healthcare policy analysis |
| 16 | Betts;1998; United Kingdom | The politics of nursing. Interview by Ian McMillan | Journal interview; Nurs Stand | Descriptive interview | Explaining the politics of nursing | Not a primary study: Interview |
| 17 | Beu et al.;2005; United States of America | Health policy issues. Nurse in Washington interns share their experiences and insight | Research article; AORN Journal | Descriptive interview | Providing a summary of lessons learned by AORN’s the Nurse in Washington Internship nurses | Not a primary study: Interview |
| 18 | Blaauw et al.;2015; South Africa | Nursing education reform in South Africa - lessons from a policy analysis study | Research article; Global Health Action | Exploratory- Descriptive Qualitative study; Policy analysis | Analyzing policy and development of the new Nursing Qualifications Framework | Healthcare policy analysis |
| 19 | Borges & Nascimento; 2004; Brazil | The nurse in the decentralization process of the health system | Research article; Revista Brasileira de Enfermagem | Exploratory- Descriptive Qualitative study; Policy analysis | The nurse’s participation in the decentralization process | Healthcare policy analysis: Process |
| 20 | Bu & Jezewski; 2007; United States of America | Developing a mid-range theory of patient advocacy through concept analysis | Research article; Journal of Advanced Nursing | Exploratory- Descriptive Qualitative study; Concept analysis | Synthesizing the concept of patient advocacy literature in nursing | Theoretical study: Related to Patient advocacy |
| 21 | Buck;2011; United States of America | Climbing up Capitol Hill and loving it: Influencing health care through legislation and advocacy (P21) | Research article; Journal of Pain and Symptom Management | Descriptive Commentary | Introducing the workshop topic about learning how to influence health care through public policy, legislation, and advocacy | Not a primary study: Commentary |
| 22 | Buckalew;1987; United States of America | Judi Buckalew: learning to play political hardball. Interview by Linda J. Pearson | Journal interview; Nurse Pract | Descriptive interview | Explaining the politics of nursing | Not a primary study: Interview |
| 23 | Burke;1998; United States of America | Nursing’s national role in health policy. Interview by Marjorie Jamieson | Journal interview; Creat Nurs | Descriptive interview | Explaining the politics of nursing | Not a primary study: Interview |
| 24 | Carr &Riesco;2007; Brazil | Rekindling of nurse-midwifery in Brazil: public policy and childbirth trends | Research article; J Midwifery Womens Health | Descriptive study | Analyzing Brazil’s Ministry of Health generated policies for childbirth services | Healthcare policy analysis |
| 25 | Chao;1993; Taiwan | Developing national health policy in Taiwan | Research article; International Nursing Review | Exploratory- Descriptive Qualitative study; | Developing national health policy | Healthcare policy development |
| 26 | CHAN et al.;1999; Hong Kong | Political participation in Hong Kong: a study | Research article; Journal of Nursing Management | Descriptive study | To investigate nurses’ level of political participation and Hong Kong their perception of political efficacy | Not qualitative study |
| 27 | Clarke; 1999; United States of America | Professional commitment and activism in the lives of five southern African American nurses | Thesis (Doctorate); Harvard University | Personal narrative methodology | The lessening of racial barriers in the nursing profession and the increased numbers of African American nurses with advanced education | History of work experiences of the nurses |
| 28 | Coates;2016; United States of America | Igniting a passion for advocacy | Journal interview; American Nurse | Descriptive interview | Explaining the nursing advocacy | Not a primary study: Interview |
| 29 | Cohen;2016; United States of America | Health Policy Repertoires and Toolboxes | Editorial Research; Policy, Politics & Nursing Practice | Descriptive Editorial | Explaining nurses’ experience that might be translated to policy | Not a primary study: Commentary |
| 30 | Colloff;1980; United States of America | Nurse political action: interview with Marge Colloff. Interview by Marion Isaacs | Journal interview; Advances in nursing science | Descriptive interview | Explaining the nurses’ political action | Not a primary study: Interview |
| 31 | Corey-Lisle et al.;1999; United States of America | Healthcare reform. Its effects on nurses | Research article; J Nurs Adm | Descriptive survey | Analyzing nurses’ perceptions of the effect of healthcare reform | Unable to access |
| 32 | Cramer;1998; United States of America | Organized Participation in Nursing: A Test of the Civic Voluntarism Model | Thesis (Doctorate); University of Nebraska | Descriptive analysis of questionnaire items | Providing the variables that affect a nurse’s decision to participate in ANA | Not nurses’ experiences of the healthcare policy intervention |
| 33 | Cruz &Marques;2006; Brazil | Elements of the Political Professional Project of the Brazilian Graduate Nurses National Association present in the Annaes de Enfermagem | Research article; Revista brasileira de enfermagem | Exploratory- Descriptive Qualitative study; Based on the historical method | Describing and characterizing the elements of the political project | Literature review: Related to politics and healthcare policy |
| 34 | Darvishpour et al.;2016; Iran | Iran’s health policymakers’ views on barriers and facilitators of nurse prescribing in their context: A qualitative study | Research article; Iranian journal of nursing and midwifery research | Descriptive qualitative study | Identifying the barriers and facilitators of nurse prescribing based on policymakers’ views in Iran | Healthcare policy analysis |
| 35 | Da Silva et al.;2016; Brazil | A policy analysis of teamwork as a proposal for healthcare humanization: implications for nursing | Research article; International Nursing Review | Exploratory- Descriptive Qualitative study; Policy analysis | Analyzing the Brazilian National Humanization Policy | Healthcare policy analysis |
| 36 | Desai;2015; United States of America | Adapting, developing and testing an instrument: For evaluation of psychometric properties of the political self-advocacy instrument in Nursing (PSAI-N) | Thesis (Doctorate); William Paterson University | Descriptive, correlational design, Reliability calculations with the examination of content validity | Testing a newly developed political self-advocacy instrument in nursing (PSAI-N) | Not nurses’ experiences of the healthcare policy intervention |
| 37 | Dickerson et al.;1994; United States of America | Interpreting political agendas from a critical social theory perspective | Research article; Nursing Outlook | Exploratory- Descriptive Qualitative study; Policy analysis | The experiences of nurses who worked to defeat the RCT proposal have changed the way many nurses view political change. | Healthcare policy analysis |
| 38 | Di Falco et al.;2021; Italy | Evaluating the effects of polices on nursing care as promoted by the Veneto region: the research protocol and its implications for public health | Research article; Annali Di Igiene Medicina Preventiva E Di Comunita | Multi-center mixed-method study design | Measuring the effect of these polices on nursing care | Effect of healthcare policy |
| 39 | Ditlopoet al.;2015;  South Africa | Contestations and complexities of nurses’ participation in policy-making in South Africa | Research article; Global Health Action | Multiple descriptive case study | Examining whether and how nurses in legislative and administrative positions advance policy or regulatory issues that are important to health care | A study on nurses’ and non-nurses’ healthcare policy intervention |
| 40 | Dobbins et al.;2007; Canada | Public health decision-makers’ informational needs and preferences for receiving research evidence | Research article; Worldviews Evid Based Nurs | Exploratory- Descriptive Qualitative study; | Identifying decision-makers preferences for the transfer and exchange of research knowledge | Healthcare policy process analysis |
| 41 | Dobbins et al.;2002; Canada | A framework for the dissemination and utilization of research for health-care policy and practice | Research article; Online Journal of Knowledge Synthesis for Nursing | Exploratory- Descriptive Qualitative study; | Constructing a comprehensive framework of healthcare research dissemination | Theoretical study: Related to research dissemination of healthcare policy |
| 42 | Donley;1979;  United States of America | A nurse’s experience in Washington... Robert Wood Johnson Health Policy Fellow | Journal interview; AORN Journal | Descriptive interview | Nurse’s experience in Washington | Not a primary study: Interview |
| 43 | Doolan;2000;  United Kingdom | Nursing. Image, politics and the media | Journal interview; Br J Perioper Nurs | Descriptive interview | Explaining the nurses’ politics, image and the media | Not a primary study: Interview |
| 44 | El-Jardali et al.;2014; Lebanon | The making of nursing practice Law in Lebanon: a policy analysis case study | Research article; Health Research Policy and Systems | Exploratory- Descriptive Qualitative study; Case study approach | Generating in-depth insights into the public policymaking process and identifying the factors that influence policymaking | Healthcare policy process analysis |
| 45 | Elder & Mitchell;1992; New Zealand | Advocacy in action. Interview by Kathy Stodart | Journal interview; N Z Nurs J | Descriptive interview | Explaining the nurses’ advocacy in action | Not a primary study: Interview |
| 46 | Etowa et al.;2016; Canada | Experiences of midwives and nurses in policy development in low- and middle-income countries: a systematic review protocol | Research article; JBI Evidence Synthesis | Systematic Review Protocol | synthesizing the qualitative evidence of nurses’ involvement in policy development | Not a primary study: Commentary |
| 47 | Fawcett & Russell;2001; United States of America | A Conceptual Model of Nursing and Health Policy | Research article; Policy, Politics, & Nursing Practice | Exploratory- Descriptive Qualitative study; Concept development | Developing of a conceptual model for nursing and health policy | Theoretical study: Related to politics and healthcare policy |
| 48 | Fealy et al.;2018; Ireland | Discursive constructions of professional identity in policy and regulatory discourse | Research article; Journal of Advanced Nursing | Exploratory- Descriptive Qualitative study; Critical discourse analysis | Examining disciplinary discourses conducted through professional policy and regulatory documents | Healthcare policy analysis |
| 49 | Fela;2018; Australia | Blood Politics: Australian Nurses, HIV and the Battle for Rights on the Wards | Research article; Labor History | Descriptive study | Nurse’s experience for HIV patient’s rights on the wards | Labor struggle in the hospital |
| 50 | Finch;2001; United States of America | Linking research to health policy: experiences of nurses in policy positions | Thesis (Doctorate); George Mason University | Exploratory- Descriptive Qualitative study; Ethnography | Exploring how to use research findings in the field of federal health policy | Theoretical study: Related to politics and healthcare policy |
| 51 | Frankel;1986; United States of America | Political prescription: more nurses. Interview by Audrey Cochran | Journal interview; California nurse | Descriptive interview | Explaining the nurses’ political prescription | Not a primary study: Interview |
| 52 | Fradd; 2004; United Kingdom | Political leadership in action | Research article; Journal of Nursing Management | Descriptive Case study | Exploring the author’s personal experience of working in National Health Service roles | History of work experiences of the nurse |
| 53 | Fuller; 2016; United States of America | The Future of Advocacy and Leadership: Faculty and Mentorship is Key | Journal interview; Florida Nurse | Descriptive interview | Explaining the future of advocacy and leadership | Not a primary study: Interview |
| 54 | Fyffe; 2009; United Kingdom | Nursing shaping and influencing health and social care policy | Research article; Journal of Nursing Management | Critically reviews | To consider how nursing profession’s role in shaping and influencing policy | Not nurses’ experiences of the healthcare policy intervention |
| 55 | Gazarian et al.;2016; United States of America | Effectiveness of narrative pedagogy in developing student nurses’ advocacy role | Research article; Nursing Ethics | Quantitative Study: Survey | Evaluating the effectiveness of narrative pedagogy on the development of advocacy in student nurses | Policy & political nursing education |
| 56 | Gilson;2010; The United States of America | Oregon’s Senate Bill 560: practical policy lessons for nurse advocates | Research article; Policy, politics & nursing practice | Exploratory- Descriptive Qualitative study; Policy analysis | Exploring competing conceptions for the SB 560 policy process | Healthcare policy analysis |
| 57 | Gleeson;2013; United Kingdom | Using policy analysis to explore the reciprocal impact of health policy on public health nursing and public health nursing on policy | Thesis (Doctorate); Bournemouth University | Exploratory- Descriptive Qualitative study; Policy analysis | Exploring the reciprocal interaction between health policy and public health nursing | Healthcare policy analysis |
| 58 | Gleeson;2015; Canada | To what extent do health visitors and school nurses have a voice in the policy process? | Research article; Community Practitioner | Exploratory- Descriptive Qualitative study; Policy analysis | Exploring the reciprocal impact of health policy on public health nursing | Healthcare policy analysis |
| 59 | Gough;1999; United Kingdom | A matter of policy. Interview by Nick Lipley | Journal interview; Nurs Stand | Descriptive interview | Explaining the matter of policy | Not a primary study: Interview |
| 60 | Hajizadeh et al.;2021; Iran | Factors influencing nurses participation in the health policy-making process: a systematic review | Research article; BMC Nursing | Systematic literature review | Identifying factors influencing nurses participation in the health policy-making process | Literature review of factors the political involvement |
| 61 | Han & Kim;2020; South Korea | Concept Development of Political Competence for Nurses | Research article; Journal of Korean Academy of Nursing | Exploratory- Descriptive Qualitative study; Using phenomenology | Defining and clarifying the concept of political competence for nurses | A study on nurses’ and non-nurses’ healthcare policy intervention |
| 62 | Hanks et al.;2018; United States of America | Patient Advocacy at the APRN Level: A Direction for the Future | Editorial Material; Nursing Forum | Descriptive literature review | Synthesizing the existing nursing of patient advocacy | Integrative literature review: Patient advocacy |
| 63 | Hartley;2013; United Kingdom | Leading with political astuteness: A study of public managers in Australia, New Zealand and the United Kingdom | Research article; Australia and New Zealand School of Government (ANZSOG) | Exploratory- Descriptive Qualitative study; Concept development | Understanding of how public managers see politics, the nature of their political astuteness | Theoretical study: Related to politics and healthcare policy |
| 64 | Haycock‐Stuart & Kean;2013; United Kingdom | Shifting the balance of care? A qualitative study of policy implementation in community nursing | Research article; Journal of Nursing Management | Mixed qualitative methods including focus groups and individual interviews | Examining the interaction among policy implementation, service organization, and delivery for community nursing services | Healthcare policy analysis: Process |
| 65 | Hofmann; 1989; United States of America | Membership status in New York State Nurses Association, political involvement and political activity of nurses, and concern with legislative issues relevant to nursing | Thesis; Columbia University | Quantitative research | Identifying the relationship between membership in the NYSNA, the Political Involvement of nurses | Not qualitative study |
| 66 | Houser; 2017; United States of America | Facilitating Political Advocacy in Baccalaureate Education Programs in a Southeastern State | Research article; Facilitating Political Advocacy in Baccalaureate Education Programs in a Southeastern State | Qualitative descriptive study | Exploring the experiences of nursing department chairperson with political advocacy in baccalaureate nursing education | Policy & political nursing education |
| 67 | Joel;1991; United States of America | ANA launches workplace initiative. SNAs get grants for RN advocacy, new organizing. Interview by Patricia McCarty | Journal interview; Am Nurse | Descriptive interview | Launching workplace initiative | Not a primary study: Interview |
| 68 | Jones;2005;  United Kingdom | Interpreting policy in the community. Interview by Carolyn Scott | Journal interview; Prof Nurse | Descriptive interview | Interpreting policy | Not a primary study: Interview |
| 69 | Joynes;1991;  United States of America | The politics of enacting prescriptive authority legislation for nurses (The Washington experience) | Conference proceeding; Clemson University College of Nursing | Conference material | Experiences of nurses’ political participation | Not a primary study:Conference material |
| 70 | Keepnews;2006;  United States of America | Bringing nursing leadership to shaping state policy: an interview with Virginia Trotter Betts | Journal interview; Policy, Politics & Nursing Practice | Descriptive interview | Using leadership in policy decision-making | Not a primary study: Interview |
| 71 | Kennedy;2000; United Kingdom | Political but not partisan. Interview by Pat Healy | Journal interview; Nurs Stand | Descriptive interview | Explaining the politics of nursing | Not a primary study: Interview |
| 72 | Kerschner & Cohen;2002; United States of America | Legislative decision making and health policy: a phenomenological study of state legislators and individual decision making | Research article; Policy, Politics & Nursing Practice | Exploratory- Descriptive Qualitative study; Phenomenology | Revealing three essential elements as forming the structure of individual legislative decision-making on health policy | Not nurses’ experiences of the healthcare policy intervention |
| 73 | Khoury et al.;2011; United States of America | Nursing Leadership From Bedside to Boardroom A Gallup National Survey of Opinion Leaders | Research article; The Journal of Nursing Administration | Descriptive interview | Examining how nursing is viewed by the nation’s decision-makers and opinion leaders | A study on non-nurses’ healthcare policy intervention |
| 74 | Kung & Lugo.; 2015; Iran | Political advocacy and practice barriers: A survey of Florida APRNs | Research article; Journal of the American Association of Nurse Practitioners | Exploratory- Descriptive Qualitative study; Grounded theory | Nurses’ patient advocacy concept in the hospital | Theoretical study: Related to patient advocacy |
| 75 | Lankarani et al.;2021; Iran | Scenarios for Dealing with the Nursing Services Tariff Setting Act Based on the Policy Analysis Triangle Mode | Research article; Sadra Medical Sciences Journal | Exploratory- Descriptive Qualitative study; Policy analysis | Making a multilateral analysis of nursing services tariff setting | Healthcare policy analysis |
| 76 | Larsson; 1974; Sweden | The nurse in civic life | Journal interview; International Nursing Review | Descriptive interview | Explaining the politics of nursing | Not a primary study: Interview |
| 77 | Leppoet al.;2013; Finland | Health in All Policies - Seizing opportunities, implementing policies | Book; Sosiaali-ja terveysministeriö | Descriptive Study; Policy analysis | Analyzing the dynamics of policymaking using Kingdon’s framework on problems, policies, politics and windows of opportunity | Healthcare Policy development |
| 78 | Lewinski & Simmons;2018; United States of America | Nurse Knowledge and Engagement in Health Policy Making: Findings From a Pilot Study | Research article; The Journal of Continuing Education in Nursing | Quantitative study; Survey | Surveying practicing nurses on their current knowledge about health policy advocacy and policy involvement | Not qualitative study |
| 79 | Logan; et al.; 2011; United States of America | Health care policy development: a critical analysis model | Research article; J Nurs Educ | Exploratory- Descriptive Qualitative study; Policy analysis | Describing a phased approach for teaching baccalaureate nursing students critical analysis of health care policy | Healthcare Policy development |
| 80 | Makhwanya & Mafalo; 2010; South Africa | The politics of International Nurses Day | Journal Letter; Nursing Update | Letter | Explaining the politics of nursing | Not a primary study: Letter |
| 81 | Malone;2000; United States of America | Beverly Malone on healthcare quality, policy, and nursing. Interview by Mary Lou Brunell and Jacqueline F. Byers | Journal interview; J Healthc Qual | Descriptive interview | Explaining the healthcare quality, policy, and nursing | Not a primary study: Interview |
| 82 | Martin;2004; United Kingdom | Policy development is paramount. Interview by Carolyn Scott | Journal interview; Prof Nurse | Descriptive interview | Explaining the politics of nursing | Not a primary study: Interview |
| 83 | Mascarenhas et al.;2021; Brazil | Nurse contribution to the construction of health policy in the state of Bahia (1925-1930) | Research article; Revista Brasileira De Enfermagem | Qualitative research, from a historical nature | Analyzing the contribution of nurses to the construction of health policy in the state of Bahia, from 1925 to 1930 | Not nurses’ experiences of the healthcare policy intervention |
| 84 | Maslin-Prothero &Masterson;2002; United Kingdom | Power, politics, and nursing in the United Kingdom | Research article; Policy, Politics & Nursing Practice | Qualitative descriptive study | Examining the effects of politics and how politics influences care and practice | Unable to access |
| 85 | Maynard;1995; United States of America | Stakeholders in the political process: nurses’ role in MinnesotaCare | Research article; Nursing Policy Forum | Descriptive study | Political activism and the roles on healthcare policy reform | Unable to access |
| 86 | McKenna et al.;2004; United Kingdom | Nurse leadership within primary care: the perceptions of community nurses, GPs, policy makers and members of the public | Research article; Journal of Nursing Management | Two-round Delphi technique; Focus group -postal questionnaire/Semi-structured interview | To seek the views of community nurses of the public and policy makers on nursing leadership in primary care | Primary care |
| 87 | McKeown & Gibson;2007; Ireland | Determining the political influence of nurses who work in the field of hepatitis C: a Delphi survey | Research article; Journal of Clinical Nursing | A multi-center survey design | Determining the political influence and the professional profile of nurses who work in the field of hepatitis C | Not nurses’ experiences of the healthcare policy intervention |
| 88 | Meira et al.;2013; Brazil | Political-ethical skill development in nursing undergraduates | Research article; Revista Da Escola De Enfermagem Da Usp | Case study | Developing curriculum in a political-ethical skill for the undergraduate nursing degree | Policy & political nursing education |
| 89 | Melo et al (2017) Brazil | Guide of attributes of the nurse’s political competence: a methodological study | Research article; Revista Brasileira de Enfermagem | - Concept development & validation and evaluation of research tools | To build and validate a guide of attributes of the nurse’s political competence | Literature review: Related to politics and healthcare policy |
| 90 | Montalvo; 2015; United States of America | Political Skill and Its Relevance to Nursing: An Integrative Review | Research article; J Nurs Adm | Exploratory- Descriptive Qualitative study; Concept analysis | Conducting an integrative analysis of literature for the concept of political skill | Literature review: Related to organization politics |
| 91 | Mund;2018; United States of America | Professional Socialization and Political Advocacy | Research article; AANA Journal | Descriptive Commentary | Explaining the Maynard Model of Political Influence | Not a primary study: Commentary |
| 92 | Nóbrega-Therrien et al.;2010; Brazil | Political pedagogical project: Conception, construction, and evaluation in nursing | Research article; Revista Da Escola De Enfermagem Da Usp | Mixed-method study design | Understanding nursing education based on the current political pedagogical projects | Policy & political nursing education |
| 93 | Nsiah et al.;2019; Ghana | Barriers to practicing patient advocacy in healthcare setting | Research article; Nursing open | Qualitative descriptive study | Exploring barriers to practicing patient advocacy in the healthcare setting | Patient advocacy in the hospital |
| 94 | O’Brien-Larivee;2011; Canada | A Service-Learning Experience to Teach Baccalaureate Nursing Students About Health Policy | Research article; Journal of Nursing Education | Descriptive study | Developing to facilitate baccalaureate nursing students’ learning about healthy public policy | Policy & Political nursing education |
| 95 | Oerther et al.;2018; United States of America | The American Academy of Nursing Jonas Policy Scholars Program: Mentoring future nurse leaders to advance health policy | Editorial Research; Journal of Advanced Nursing | Descriptive Editorial | Discussing the importance of mentoring nurse leaders for advancing health policy | Not a primary study: Commentary |
| 96 | Oestberg;2012; United States of America | Policy and Politics: Why nurses should get involved | Journal i pictorial; Nursing | pictorial | Explaining the politics of nursing | Not a primary study: pictorial |
| 97 | ONZM et al.;2006; New Zealand | Reconnecting with policy - Requirements for survival as a mental health nurse | Research article; Journal of Psychosocial Nursing and Mental Health Services | Descriptive research | Discussing the disconnection between mental health nurses and policy | Not a primary study: Perspective |
| 98 | Pal & Clark;2015; Canada | Making reform stick: Political acumen as an element of political capacity for policy change and innovation | Research article; Policy and Society | Descriptive study | Focusing on policy capacity for significant change and/or innovation at the system level | Not nurses’ experiences of the healthcare policy intervention |
| 99 | Pamela;2011; Canada | Intersection of National Policies on Nurses’ Work in District Health Care Systems in Kenya | Thesis (Doctorate); University of Ottawa | Multi-center mixed-method study design | Examining how the national policy reforms have impacted nurses’ work | A study on nurses’ and non-nurses’ healthcare policy intervention |
| 100 | Peacock & Brennan;1992; United States of America | Nurses affecting change; enhancing our image through healthcare reform | Letter; ANNA Journal | Letter | Expanding role of the nurse in the formulation of public policy | Unable to access |
| 101 | Peruzzo et al.;2006; Brazil | The Brazilian Nursing Political Professional Project and the Aben-Paraná presidents between 1980 and 2001 | Research article; Revista brasileira de enfermagem | Qualitative study based on the historical method | Historicizing the development of the Brazilian Nursing Political-Professional Project | Not a primary study: Perspective |
| 102 | Peters;2019; Australia | Nurse and carer experiences of working in aged care: research for policy impact | Research article; Australian Nursing & Midwifery Journal | Quantitative research: Survey | Focusing on the importance of the participation in research of nurses and carers working in aged care | Not qualitative study |
| 103 | Phillips;2012; United States of America | Nurses Becoming Political Advocates | Research article; Journal of Emergency Nursing | Descriptive Commentary | Following literature review sought information on the components of nursing political advocacy | Not a primary study: Commentary |
| 104 | Primomo & Björling;2013; United States of America | Changes in Political Astuteness Following Nurse Legislative Day | Research article; Policy, Politics, & Nursing Practice | Quantitative research: Survey | Determining if political astuteness changed after participants attended a state nurse legislative day | Not qualitative study |
| 105 | Rainer;2015; United States of America | Speaking Up Factors and Issues in Nurses Advocating for Patients When Patients Are in Jeopardy | Research article; Journal of Nursing Care Quality | Exploratory- Descriptive Qualitative study; Concept development | Conducting a literature review and offering a new theoretical framework | Theoretical study: Related to patient advocacy |
| 106 | Ramesh et al.;2016; Singapore | Measuring individual-level analytical, managerial and political policy capacity: A survey instrument | Research article; Lee Kuan Yew School of Public Policy Research Paper | Exploratory- Descriptive Qualitative study; Measuring tool development | Discussing the development of instrument to address this gap in public policy literature | A study on non-nurses’ healthcare policy intervention |
| 107 | Rasheed et al.;2020; Pakistan | Challenges, Extent of Involvement, and the Impact of Nurses’ Involvement in Politics and Policy Making in in Last Two Decades: An Integrative Review | Research article; Journal of Nursing Scholarship | Integrative literature review | Determining nurses’ challenges, the extent of involvement, and the impact of involvement in policy-making | Literature review of political involvement |
| 108 | Rezende et al.; 2020; Brazil | Nurses’ practice in quilombola communities: an interface between cultural and political competence | Research article; Revista Brasileira De Enfermagem | Qualitative case study | Understanding Family Health Strategy nurses’ practices in the communities for political competencies | Primary Health care |
| 109 | Rezende et al.;2021; Brazil | The practice of nurses in the implementation of public policies for the black population: in the light of feminist ethics | Research article; Saude E Sociedade | Qualitative study | Understanding FHS nurses’ practices in the context of quilom bola communities for political competencies. | Primary Health care |
| 110 | Ridenour & Trautman;2009; United States of America | A Primer for Nurses on Advancing Health Reform Policy | Research article; Journal of Professional Nursing | Discriptive study | Presenting an overview of health reform, provide insight into the process and dimensions | A Primer for Nurses on participating in politics or policy intervention |
| 111 | Rubotzky;1999; United States of America | An oral history of nursing participation in the healthcare reform efforts of 1993-1994 | Thesis (Doctorate); University of Colorado | Qualitative descriptive Oral history: Based on postmodernism and feminism | Creating of the experiences of thirteen women who held leadership positions in the healthcare reform | A study on nurses’ and non-nurses’ healthcare policy intervention |
| 112 | Safari et al.;2020; Iran | The Related Factors of Nurses’ Participation and Perceived Benefits and Barriers in Health Policy-Making | Research article; Journal of Nursing Research | Cross-sectional descriptive study | Examining the factors related to the participation in health policymaking, benefits and barriers | Not qualitative study |
| 113 | Salhani &Coulter;2009; Canada | The politics of interprofessional working and the struggle for professional autonomy in nursing | Research article; Social Science & Medicine | Exploratory- Descriptive Qualitative study; Ethnographic study | Realizing nursing’s professional project and how the politics of professional autonomy | Nursing advocacy in the hospital |
| 114 | Sessler;2012; United States of America | The Nurse as Advocate: A Grounded Theory Perspective | Thesis (Doctorate); Villanova University | Exploratory- Descriptive Qualitative study; A Grounded Theory | Increasing the current knowledge about the nurse advocate role | Theoretical study: Related to advocacy model |
| 115 | Scott;2004; United Kingdom | It’s my job to challenge and shape policy. Interview by Carolyn Scott | Journal interview; Prof Nurse | Descriptive interview | Explaining the politics of nursing | Not a primary study: Interview |
| 116 | Sells et al.;2018; United States of America | Engaging in Policy During Graduate Training | Research article; Annual Review of Nursing Research | Descriptive study | Exploring the experiences of a Doctor of Nursing Practice as they integrate public policy education | Policy & political nursing education |
| 117 | Sharp;1994; United States of America | An analysis of lobbying strategies used to influence health policy | Thesis (Doctorate); George Mason University | Descriptive study | Analyzing lobbying strategies used by lobbyists hired by special interest groups to influence health | Healthcare policy analysis: Process (Healthcare Staffs) |
| 118 | Sharp;2003; United States of America | Health policy internships and fellowships in Washington, DC | Promotional material; Newborn & Infant Nursing Reviews | Education program promotiona | Creating a special internship to meet the nurse’s needs | Not a primary study: Education program promotion |
| 119 | Shatz;2018; United States of America | A Politics of Care: Local Nurses in Mandate Palestine | Research article; International Journal of Middle East Studies | Qualitative study based on the historical method | Examining the work experiences of Palestinian Arab nurses to illuminate the operation of the colonial public health regime | History of work experiences of the public healthcare policy |
| 120 | Shuai & Macduff;2016; Scotland | An analysis of policy to practice initiatives in Scotland: what are the key lessons learned? | Research article; Journal of Nursing Management | Multiple case study design | Identifying common issues and lessons arising from four national health policy initiatives related to nursing | A study on non-nurses’ healthcare policy intervention |
| 121 | Schutzenhofer & Cannon; 1986; United States of America | Moving nurses into the political process | Research article; Nurse Educator | Descriptive study | Offering nurses’ political involvement to develop their political competence | Policy & political nursing education |
| 122 | Sochalski & Pulcini;2014; United States of America | Interview with a Nursing Policy Leader: A Hopeful Look at a Changing Profession | Journal interview; AJN The American Journal of Nursing | Descriptive interview | Explaining the politics of nursing | Not a primary study: Interview |
| 123 | Spitzer-Lehmann; 1995; United States of America | The politics of advanced practice: an interview with Roxane Spitzer-Lehmann | Journal interview; Advanced Practice Nursing Quarterly | Descriptive interview | Explaining the politics of nursing | Not a primary study: Interview |
| 124 | Squellati;2018; United States of America | A Policy Apprenticeship in the Office of U.S. Senator Daniel K. Inouye | Research article; Annual Review of Nursing Research | Descriptive study | Learning as an apprentice from the Senator and policy-making process | Policy & political nursing education |
| 125 | Taft & Nanna;2008; United States of America | What Are the Sources of Health Policy That Influence Nursing Practice? | Research article; Policy, Politics, & Nursing Practice | Exploratory- Descriptive Qualitative study | Proposing a framework of the sources of health policy | Theoretical study: Related to politics and healthcare policy |
| 126 | Thomas et al.;2020; United States of America | How to Engage Nursing Students in Health Policy: Results of a Survey Assessing Students’ Competencies, Experiences, Interests, and Values | Research article; Policy, Politics & Nursing Practice | Quantitative Study: Survey | Assessing nursing students’ perceptions of health policy competencies | Policy & political nursing education |
| 127 | Thompson et al.;2005; United States of America | Policy perspectives of major nursing organizations, part II. Interview by David M Keepnews | Research article; Policy, politics & nursing practice | Descriptive interview | Presenting interviews about organizations’ policy priorities and policy-related activities | Not a primary study: Perspective |
| 128 | Towers; 1998; United Kingdom | Virginia Trotter Betts, MSN, JD, RN, FAAN, Senior Advisor on Nursing and Policy to the Secretary and Assistant Secretary of Health and Human Services. Interview by Jan Towers and Carole Jennings | Journal interview; J Am Acad Nurse Pract | Descriptive interview | Explaining the politics of nursing | Not a primary study: Interview |
| 129 | Triolo;1986; United States of America | Certified nurse-midwife political involvement: regulatory and licensing board | Research article; Journal of Nurse-Midwifery | Descriptive study | Reviewing one Certified nurse-midwife (CNM’s) experience with the executive branch of state government | A primer for nurses on participating in politics or policy intervention |
| 130 | Vaartio & Leino-Kilpi.;2005; Finland | Nursing advocacy: a review of the empirical research 1990-2003 | Research article; International Journal of Nursing Studies | Descriptive literature review | Overview of the research literature on nursing advocacy | Integrative literature review: Nursing advocacy |
| 131 | Vandenhouten et al.;2011; United States of America | Political Participation of Registered Nurses | Research article; Policy, Politics, & Nursing Practice | A descriptive, predictive design; Based on the CVM (Verba et al., 1995) | Measuring of Level of political participation and factors contributing to participation | Not qualitative study |
| 132 | Van Hoover;2019; United States of America | How to Run for Political Office: A Primer for Midwives and Other Nontraditional Candidates | Research article; J Midwifery Womens Health | Descriptive study | Providing of a practical guide to the basics of running for local office | A primer for nurses on participating in politics or policy intervention |
| 133 | Wall;2013; United States of America | The role of Catholic nurses in women’s health care policy disputes: A historical study | Research article; Nursing Outlook | Descriptive study | Examining the role of Catholic sister nurses in health policy for women | History of nurses’ roles in the hospital |
| 134 | Waring et al.;2018; United Kingdom | Healthcare leadership with political astuteness (HeLPA): a qualitative study of how service leaders understand and mediate the informal ‘power and politics’ of major health system change | Research article; BMC health services research | a qualitative narrative methodology: in depth ethnographic research | Investigating the acquisition, use and contribution of political ‘astuteness’ in the implementation of strategic health system change | A study on non-nurses’ healthcare policy intervention |
| 135 | Warren;2010; Canada | Nurses: influencers of healthcare policy: a master’s student reflects on her experience at NANB | Research article; Info Nursing | Quantitative Study | The factors of healthcare policy | Policy & political nursing education |
| 136 | Whelan;1995; United Kingdom | Time to get tougher... nurses have to become more political | Research article; Editorial | Descriptive Editorial | Explaining the politics of nursing | Not a primary study: Commentary |
| 137 | Williams;1993; Brazil | Commentary on Community health learning experiences and political activism: a model for baccalaureate curriculum revolution content | Research Commentary; Journal of Nursing Education | Descriptive Commentary | Describing rationales and advocating political activism as a model for baccalaureate nursing programs | Not a primary study: Commentary |
| 138 | Williamson & Prosser;2002; United Kingdom | Action research: politics, ethics and participation | Research article; J Adv Nurs | Descriptive study | Understanding of the political and ethical aspects of action research | Not a primary study: Review |
| 139 | Wilson;1997; United States of America | Nursing effects in policy-making | Thesis (Doctorate);Union Institute | Exploratory- Descriptive Qualitative study; Phenomenology | Exploration of nursing effects, in three broad categories: education, ‘professional politics,’ and health care | A study on non-nurses’ healthcare policy intervention |
| 140 | Winter & Lockhart;1993; United States of America | Understanding the political involvement of nurses: a phenomenological investigation with implications for nursing education | Research article; Pennsylvania Nurse | Phenomenology Research | Exploring the experiences of nurses’ political involvement in nursing education | Policy & political nursing education |
| 141 | Wold et al.;2004; United States of America | Teaching the public health core competency of policy development to baccalaureate student nurses | Research article; Family & Community Health | Descriptive study | Teaching the public health core competency to baccalaureate student nurses | Policy & political nursing education |
| 142 | Wolf; 2017; Germany | Political participation in times of bologna and social web – A grounded theory from a students’ point of view | Conference proceeding; E-Democracy and Open Governement | Conference material | Activation of political participation using social web and bologna | Not a primary study: Conference material |
| 143 | Wu & Howlett;2015; Singapore | Policy capacity: A conceptual framework for understanding policy competences and capabilities | Research article; Policy and Society | Exploratory- Descriptive Qualitative study; Concept development | Presenting a conceptual framework for analyzing and measuring policy capacity under which policy capacity refers to the competencies and capabilities important to policy-making | Theoretical study: Related to politics and healthcare policy |
| 144 | Yoho et al.;2004; United States of America | Student perspectives on the George Mason University (GMU) 12th annual Washington Health Policy Summer Institute | Conference proceeding;Policy, Politics & Nursing Practice | Conference material: Pictorial | the experiences of doctoral students who participated in GMU Washington Health Policy Institute | Not a primary study: Conference material |
| 145 | Yurdin;2008; United States of America | Nursing that works. Bridging the gap between organizational policies and practice. Interviewed by Diane E Scott | Research article; Fla Nurse | Descriptive interview | Providing the perspective of bridging between organizational policies and practice | Not a primary study: Perspective |
| 146 | Hajizadeh et al.; 2021; Iran | Factors influencing nurses participation in the health policy-making process: a  systematic revie | Research article; BMC Nursing | Systematic Review | Identifying factors influencing nurses participation in  the health policy-making process | Not a primary study: Systematic Review |
| 147 | Han & Lee; 2022; South Korea | A policy review of the process of the Integrated Nursing Care Service System in South Korea | Research article; Journal of Nursing Management | Policy review | Analysing the policy formation process of South Korean Integrated Nursing Care Service System | Not a primary study: Policy review |
| 148 | Safari et al.; 2020; Iran | The Related Factors of Nurses’ Participation and Perceived Benefits and Barriers in Health Policy Making | Research article; The Journal of Nursing Research | Cross-sectional descriptive study | Examining the factors related to the participation of nurses in the provision of health services and the perceived benefits and barriers to their participation in health policy making | Not qualitative study |
| 149 | Etowa et al.;2023; Canada | Experiences of nurses and midwives in policy development in low- and middle-income countries: Qualitative systematic review | Research article; International Journal of Nursing Studies Advances | Systematic Review | Identifying appraise and synthesize the qualitative evidence on the experiences of nurses’ and midwives’ involvement in policy development LMICs. | Not a primary study: Systematic Review |
