# Supplementary material for: The Barriers and Facilitators Influencing Nurses' Political Participation or Healthcare Policy Intervention: A Systematic Review and Qualitative Meta-Synthesis
Source: J Nurs Manag. 2024 Jun 28;2024:2606855. doi: 10.1155/2024/2606855 (PMC11919103; doi:10.1155/2024/2606855)
Supplement: Supplementary Materials — include seven files that provide further information about search strategies, excluded articles based on the full-text review, the PRISMA 2020 checklist, a list of the selected articles for analysis, the findings (barriers and facilitators), the GRADE CERQual assessment, and the eMERGEe reporting result. [file 2606855.f1.zip › 4_Selected articles list.docx]

**Supplementary table 4. Selected articles list for analysis**

| The 18 studies included in the analysis were conducted in the United States (n = 7), Brazil (n = 2), Ghana (n = 2), Iran (n = 2), New Zealand (n = 1), Canada (n = 1), South Korea (n = 1), Thailand (n = 1) and Kenya (n = 1). |
| --- |

[7] Hajizadeh, A., Zamanzadeh, V., & Khodayari-Zarnaq, R. Exploration of knowledge, attitudes, and perceived benefits towards nurse managers’ participation in the health policy-making process: a qualitative thematic analysis study. *Journal of Research in Nursing*, *27*(6), 560-571, 2022.

[10] A. Hajizadeh, V. Zamanzadeh and R. Khodayari‐Zarnaq, “Participation of nurse managers in the health policy process: A qualitative study of barriers and facilitators,” International Nursing Review, vol. 68, no. 3, pp. 388–398, 2021.

[11] Han, N. Korean nurses’ participation in health care policy reform: A phenomenological study,” Journal of Nursing Management, vol.28, pp. 1347–1355, 2020.

[13] J. E. Deschaine and M. A. Schaffer, “Strengthening the role of public health nurse leaders in policy development,” Policy, Politics & Nursing Practice, vol. 4, no. 4, pp. 266–274, 2003

[14] M. L. Dollinger, A grounded theory study of nurse advocacy in health policy. State University of New York at Buffalo, Location, 2007.

[26] A. K. Acheampong, L. A. Ohene, I. N. A. Asante, et al., “Nurses’ and midwives’ perspectives on participation in national policy development, review and reforms in ghana: A qualitative study,” BMC Nursing, vol. 20, no. 1, pp. 26, 2021.

[27] C. T. Barry, A descriptive study of the political socialization processes of nurses in specialized roles in the federal and state governments. George Mason University, Location, 1989.

[28] K. M. DiGaudio, Nurses’ participation in policy making activities. State University of New York at Buffalo, Location, 1993.

[29] D. J. Donovan, D. Diers and J. Carryer, “Perceptions of policy and political leadership in nursing in New Zealand,” Nursing Praxis in New Zealand, vol. 28, no. 2, pp. 15–25, 2012.

[30] Laari, L., & Duma, S. E. Barriers to nurses health advocacy role. *Nursing ethics*, *30*(6), 844-856, 2023.

[31] Melo, C. M. M. D., & Santos, T. A. D. Nurse's political participation in municipal Public Health Care System management. *Texto & Contexto-Enfermagem*, *16*, 426-432, 2007

[32] Rabelo, A. R. M., & Silva, K. L. D. Let it not be that nursing that asks for silence: participation in social movements and sociopolitical-emancipatory knowledge. *Revista Brasileira de Enfermagem*, *75*, e20210630, 2022.

[33] N. Jivraj Shariff, “A delphi survey of leadership attributes necessary for national nurse leaders’ participation in health policy development: An East African perspective,” BMC Nursing, vol. 14, no. 1, pp. 13, 2015.

[34] M. R. Taylor, “Impact of advocacy initiatives on nurses’ motivation to sustain momentum in public policy advocacy,” Journal of Professional Nursing, vol. 32, no. 3, pp. 235–245, 2016.

[35] J. R. Warner, “A phenomenological approach to political competence: Stories of nurse activists,” Policy, Politics & Nursing Practice, vol. 4, no. 2, pp. 135–143, 2003.

[36] O. Wichaikhum, K. Abhicharttibutra, A. Nantsupawat, Y. Kowitlawakul and W. Kunaviktikul, “Developing a strategic model of participation in policy development for nurses,” International Nursing Review, vol. 67, no. 1, 11–18, 2020.

[37] T. T. Williams, Political advocacy in nursing: Perspectives from the field. The University of Alabama, Location, 2018.

[38] Wilson, D. M., Underwood, L., Kim, S., Olukotun, M., & Errasti-Ibarrondo, B. How and why nurses became involved in politics or political action, and the outcomes or impacts of this involvement. *Nursing outlook*, *70*(1), 55-63, 2022.
