# Supplementary material for: The Barriers and Facilitators Influencing Nurses' Political Participation or Healthcare Policy Intervention: A Systematic Review and Qualitative Meta-Synthesis
Source: J Nurs Manag. 2024 Jun 28;2024:2606855. doi: 10.1155/2024/2606855 (PMC11919103; doi:10.1155/2024/2606855)
Supplement: Supplementary Materials — include seven files that provide further information about search strategies, excluded articles based on the full-text review, the PRISMA 2020 checklist, a list of the selected articles for analysis, the findings (barriers and facilitators), the GRADE CERQual assessment, and the eMERGEe reporting result. [file 2606855.f1.zip › 5_FIND~1.DOC]

**Supplementary table 5. The results of synthesis findings**

After analyzing the quotes from the individual papers in this study, 232 findings were derived: 114 barriers and 118 facilitators of nurses’ political participation or involvement in health policy. The findings were then synthesized into categories of personal, organizational, and professional factors, resulting in eight themes and 22 subthemes for barriers and seven themes and 21 subthemes for facilitators.

**1. Barriers of political participation or policy intervention**

| **Themes** | **Subthemes** | **Findings** | **References** |
| --- | --- | --- | --- |
| **Personal factors** | | | |
| Lack of political interest of nurses | Gap in political beliefs and values | Influence of family background on activism | [27, 13, 28, 34, 37] |
|  |  | Lack of nurses with a sense of problem-solving or social responsibility within the limitations of the healthcare system | [7, 28, 14, 13, 11, 34, 37] |
|  |  | Lack of recognition of professionalism and nursing identity as a nursing profession | [7, 26, 28, 14, 11, 34, 37] |
|  |  | Lack of recognition of pride in the nursing profession | [26, 28, 14, 11, 34, 37] |
|  |  | Educational and socioeconomic disparity among nurses | [26, 28, 14, 34, 37] |
|  |  | Lack of Self-Valuation and Representation | [38] |
|  | Lack of political efficacy | Lack of interest and participation in political participation or policy intervention | [7, 28, 14, 10, 11, 34, 37, 38] |
|  |  | Nurses' perceived lack of impact in policy-making processes | [7, 26, 28, 14, 10, 11, 34, 37] |
|  |  | Fear of speaking out and professional consequences | [26, 28, 10, 34, 37] |
|  |  | Disagreement with nurses' perception of lack of recognition | [26, 28, 10, 34, 37] |
|  |  | Lack of political recognition | [26, 28, 14, 10, 11, 34, 37] |
|  |  | Fear of reactions and consequences in policy intervention | [26, 10, 34, 37] |
|  |  | Lack of challenges in political awareness | [26, 28, 14, 10, 11, 34, 37] |
|  |  | Lack of awareness of the need for health policy development | [26, 28, 14, 10, 11, 34, 37] |
| Lack of political competence of nurses | Lack of political knowledge and information | Lack of knowledge necessary to navigate the political landscape and effectively influence policy decisions in favour of public health | [7, 27, 13, 28, 14, 29, 10, 11, 34, 37, 38] |
|  |  | Educational gaps in policy knowledge among nurses | [7, 27, 13, 28, 14, 29, 10, 34, 37] |
|  | Lack of political skill | Competency gaps in political leadership and management | [13, 28, 14, 29, 10, 11, 34, 37] |
|  |  | Limitations of political leadership roles | [13, 28, 29, 10, 11, 34, 37] |
|  |  | Lack of motivation to acquire political skills due to the long-term nature of political advocacy | [28, 14, 29, 10, 11, 34, 37] |
|  |  | Lack of patience required in the political advocacy process | [28, 29, 10, 11, 34, 37] |
|  | Lack of participation in activities of nursing representative organizations | Lack of awareness of professional association activities among nurses | [28, 31, 34, 37] |
|  |  | Lack of self-valuation and representation | [28, 34, 37] |
|  |  | Lack of participation in nursing representative organizations’ activities | [28, 38, 34, 37] |
|  | Lack of political participation | Lack of challenges in political engagement of nurses | [7, 26, 28, 14, 29, 10, 11, 34, 37, 38] |
|  |  | Lack of various political activities of nurses | [7, 26, 28, 14, 29, 10, 11, 34, 37, 38] |
|  | Lack of awareness of the policy process | Policy: Barriers to national policy participation | [26, 28, 14, 29, 10, 11, 34, 37] |
|  |  | Exclusion and recognition in policy development | [26, 28, 14, 29, 10, 11, 34, 37] |
|  |  | Lack of challenges in political engagement among nurses | [26, 28, 14, 29, 10, 11, 34, 37] |
|  |  | Limited nurse involvement in policy development and decision-making | [7, 26, 28, 14, 29, 10, 11, 34, 37, 38] |
| **Organizational factors** | | | |
| Nurses’ working environment constraints | Poor working environments | Nurses' lack of interest in political participation because of bad working conditions | [26, 28, 10, 11, 32, 34, 37] |
|  |  | Nurses are frequently working overtime and night shifts, which leads to labour intensity issues | [26, 28, 10, 11, 34, 37] |
|  |  | Nurses' perception of poor working conditions | [26, 28, 10, 11, 34, 37] |
|  |  | Difficulties in political participation due to lack of time due to nurses working shifts | [26, 28, 11, 34, 37] |
|  |  | Disparities in wages and benefits across healthcare organizations | [26, 28, 34, 37] |
|  |  | Perception that political engagement is not conducive to nurses' career development | [26, 28, 10, 34, 37] |
|  | Time and resource constraints | Time and resource constraints and workload issues due to the impact of nurses' work environment | [26, 28, 14, 10, 11, 34, 37] |
|  |  | Lack of time for political engagement due to juggling parenting and work | [26, 28, 34, 37] |
|  |  | Low salary in healthcare organizations leads to lack of nurse Engagement | [26, 28, 34, 37] |
| Barrers to organizational culture | Nepotism and favoritism within the organization | Challenges of nepotism and favouritism in healthcare institution | [26] |
|  |  | Nursing practice: Lack of underemphasis on advocacy in practice | [26, 28, 34, 35,37] |
|  | Hierarchical and structural organizational culture | Institutional hierarchies limiting nurse involvement | [26, 28, 10, 34, 37, 38] |
|  |  | Lack of institutional recognition of nursing roles | [26, 28, 10, 34, 37] |
|  |  | Conservative governance in achieving full support for initiatives | [26, 28, 10, 30, 32, 34, 37] |
|  |  | Organizational structure: fear of reprisal | [26, 28, 10, 34, 37] |
|  |  | Perception of disunity among nurses and healthcare profession | [26, 28, 10, 34, 37] |
|  |  | Deprivation of opportunities for nurses who speak out in policy development | [26, 28, 10, 34, 37] |
|  |  | Instability of respect and status in professional relationships within healthcare organizations | [26, 28, 10, 34, 37] |
|  | Generation differences among nurses | Generational differences in political participation | [27, 28] |
|  |  | Large age gap between nurses leads to generational differences in perceptions of politics | [27, 28] |
| **Professional factors** | | | |
| Stereotypes of the nursing profession | Undervaluation of nurses' expertise | Perception of nurses as lacking knowledge in policy development by political arena | [7, 26, 28, 14, 29, 30, 10,34, 37] |
|  |  | Comparison of nurses' intelligence and knowledge to other healthcare workers | [26, 28, 14, 29, 10, 34, 37] |
|  |  | Nurses’ facing professional repercussions if they assert themselves in policy discussions | [26, 28, 14, 29, 10, 34, 37] |
|  |  | Nurses' reluctance to participate in policy development due to perceived lack of knowledge and importance | [26, 28, 14, 29, 10, 34, 37] |
|  |  | Public and official misunderstanding of public health | [26, 28, 14, 29, 34, 37] |
|  | Gender biases | Historical antecedents’ perception of nursing as a subservient role due to historical image and gender stereotypes | [26, 13, 28, 34, 37] |
|  |  | Perception of nursing as a subservient role due to historical image and gender stereotypes | [26, 13, 28, 34, 37] |
|  |  | Gender bias and lack of respect for females in the nursing profession | [26, 13, 28, 30, 32, 34, 37, 38] |
|  |  | Fear of speaking out and professional consequences | [26, 28, 34, 37] |
|  |  | Gender dynamics in policy decision-making | [26, 13, 28, 34, 37, 38] |
| Lack of political nursing education | Insufficient education and training in political competence | Lack of formal or informal political nursing education and underemphasis on advocacy in nursing education | [7, 26, 27, 28, 14, 29, 10, 11, 30, 31, 34, 35, 37, 38] |
|  |  | Reliance on informal political skill development | [26, 27, 28, 14, 29, 10, 11, 30, 31, 34, 37] |
|  |  | Nursing education: lack of training and education | [7, 26, 27, 28, 14, 29, 10, 11, 30, 31, 34, 37, 38] |
|  |  | Nursing education: Lack of interesting in public policy | [26, 27, 28, 14, 29, 10, 11, 34, 37] |
|  |  | Need for policies to strengthen collaboration between clinical settings and educational institutions | [28, 29, 10, 11, 34, 37] |
|  |  | Lack of political socialization through formal nursing education | [26, 27, 28, 14, 29, 10, 11, 34, 37] |
|  |  | Overcrowding and impact on educational quality | [26, 28] |
|  |  | Lack of political manipulation in nursing education | [26, 28] |
|  |  | Possibility of nepotism intervention in student selection | [26, 28] |
|  |  | lack of genuine passion for the nursing education | [28, 10, 34, 37] |
|  |  | Underemphasis on advocacy in nursing education | [26, 27, 28, 14, 29, 10, 11, 34, 37] |
|  |  | Lack of formal advocacy political skill development training process | [26, 27, 28, 14, 29, 10, 11, 34, 37] |
|  |  | Need for policies to strengthen collaboration between clinical settings and educational institutions | [26, 27, 28, 10] |
|  |  | Dependency on specific individuals | [28, 34, 37] |
|  |  | Necessity of imitations other profession' political education in utilizing nursing education | [28, 34, 37] |
|  |  | Importance of further education and training for nurses in policy development | [26, 27, 28, 29, 10, 11,34, 37] |
|  | Lack of mentorship and legislative internship | Nursing education: importance of experienced and skilled tutors in nursing education | [7, 26, 28, 29, 10, 11, 34, 37] |
|  |  | Nursing education: impact of inexperienced tutors/lecturers on student nurse training | [26, 28, 29, 10, 34, 37] |
|  |  | Lack of succession planning for nurturing nursing political activists | [28, 29, 10, 34, 37] |
|  |  | Dependency on specific individuals (nurse activists) | [26, 28, 29, 10, 34, 37] |
|  |  | Lack of succession planning: very minimal informal mentorship | [26, 28, 29] |
|  |  | Lack of succession planning: queen bee syndrome' in Leadership | [28, 29] |
|  |  | Need for mentorship and advice for political activity by political nursing activists or nursing organizations | [28, 29, 10, 34, 37] |
| Limited of support for nurses by nursing representative Organizations | Insufficient advocacy efforts for nurses | Lack of awareness of association efforts and social issues | [7, 28, 34, 35, 37, 38] |
|  |  | Absence of infrastructure for monitoring advocacy skills | [28, 34, 37] |
|  |  | Lack of call to action for greater use of power to nurse by nursing representative organizations | [28, 34, 37] |
|  | Deficiency of political resources and minimal encouragement for political participation | Lack of opportunities to utilize nursing experience in political activism or healthcare policy interventions | [28, 29, 10, 34, 37] |
|  |  | Lack of responsiveness or support for nurses' needs from nurse representative organizations | [28, 34, 37] |
|  |  | Limitation in infrastructure for political skill Development for nurses | [28, 34, 37, 38] |
|  |  | Political considerations and funding instability | [28, 34, 37] |
|  |  | Limited resources and tools: Lack of resources and tools for effective policy work | [28, 10, 34, 37] |
| Lack of political power of nursing representative organizations | Lack of political network-building and communication skills | Collaborative nature of policy development | [28, 10, 11, 34, 35, 37] |
|  |  | Limitations imposed by misperceptions about networking | [28, 10, 11, 34, 35, 37] |
|  |  | Isolation of nursing from broader health system | [28, 34, 37] |
|  |  | Perceived discomfort with networking among nurses | [28, 34, 35, 37] |
|  |  | Lack of building social solidarity | [28, 34, 37] |
|  | Lack of conflict management capabilities among interest groups | Perception of asking for help as weakness | [28, 11, 35] |
|  |  | Dominance of doctors in policy development teams | [26, 28, 14, 10, 11, 34, 37] |
|  |  | Conflicts and challenges of overlapping role among healthcare profession | [28, 29, 34, 37] |
|  |  | Lack of effective negotiation skills with interest groups | [28, 10, 34, 37] |
|  |  | Lack of effective communication skills with interest groups | [28, 34, 37] |
|  | Interaction barriers with legislators and difficulty articulating nursing perspectives | Limitations in political advocacy by nursing representative organizations | [26, 28, 14, 11, 34, 35, 37] |
|  |  | Oppression theory in healthcare: Devaluing the nursing profession | [26, 28, 14, 10, 11, 34, 37] |
|  |  | Lack of effective communication skills with politicians and legislators | [28, 29, 10, 34, 35, 37] |
|  | Insufficient intervention in policy decision-making processes | Periods of inactivity and frustration in policy environment | [28, 34, 35, 37] |
|  |  | Frustration with slow policy-making processes | [28, 34, 37] |
|  |  | Lack of policy intervention in healthcare policy-making process by nursing representative organizations | [7, 26, 28, 14, 10, 11, 32, 34, 37, 38] |
|  |  | Challenges in communication with legislators | [28, 29, 11, 34, 37] |
|  |  | Challenges in advocacy communication | [29, 10, 34, 37] |
|  |  | Lack of transparency in policy development by nurses | [26, 28, 29, 10, 34, 37] |
|  |  | Policy environment: Limited time and resources | [26, 28, 11, 34, 37] |
|  |  | Exclusion from policy development of nursing profession | [26, 28, 14, 29, 10, 34, 37] |
|  |  | Barriers to participation in national policies | [26, 28, 14, 29, 10, 11, 34, 37] |
|  |  | Need for legitimate recognition of nurses' role in policy development | [26, 28, 29, 10, 34, 37] |
|  |  | Individual policy makers: Barriers to enacting public health policies, potentially due to differing priorities or perspectives | [26, 28, 29, 10, 11, 34, 37] |

**2. Facilitators to political participation or policy intervention**

| **Themes** | **Subthemes** | **Findings** | **References** |  |  |  |
| --- | --- | --- | --- | --- | --- | --- |
| **Personal factors** | | | |  |  |  |
| Recognition of social responsibilities | Enhancing nursing professional values | Clarity or sharing of nursing values and vision | [7, 28, 30, 31, 11, 33, 34, 35, 37, 38] |  |  |  |
|  |  | Increasing the sustainability of advocacy efforts through political passion | [28, 11, 31, 34, 35, 37, 38] |  |  |  |
|  |  | Recognizing social responsibility to advocate for public health and the rights of nurses | [28, 11, 31, 32, 34, 35, 36, 37] |  |  |  |
|  |  | Focus on gains rather than setbacks Acknowledgment of small gains amidst obstacles | [38] |  |  |  |
|  |  | Strengthening nursing professional pride, identity, and nurses' natural aptitude for politics | [28, 11, 14, 34, 35, 37] |  |  |  |
|  | Awareness of healthcare problems | Increasing awareness of health policy issues and self-awareness as a nursing profession | [27, 28, 29, 10, 11, 31, 33, 34-37] |  |  |  |
|  |  | Increasing political interest | [7, 28, 14, 29, 10, 11, 14, 30-37] |  |  |  |
|  |  | Activating political awareness political expression | [28, 29, 11, 33, 34, 35, 37, 38] |  |  |  |
|  |  | Boosting passion and motivation for political engagement | [28, 14, 29, 10, 11, 31, 34, 35, 37] |  |  |  |
|  |  | Nurses as advocates for vulnerable populations | [28, 11, 34, 37] |  |  |  |
|  |  | Improving observational skills and quick decision-making for healthcare issues | [28, 29, 11, 34, 35, 37] |  |  |  |
|  |  | Increasing versatility and ability to handle complex healthcare issues | [28, 29, 11, 33-37] |  |  |  |
|  |  | Becoming politician_exposure to social Injustices | [38] |  |  |  |
|  |  | Enhancing problem-solving skills and understanding of complex policy issues | [29, 33, 10, 11, 34, 35, 37] |  |  |  |
| Enhancing nurses’ political competence | Accumulating of political knowledge, information, and skills | Promoting nursing professionalism: Political knowledge and information | [7, 27, 13, 28, 14, 29, 11, 31-37] |  |  |  |
|  |  | Enhancing political skills such as communication and negotiation skills | [7, 27, 28, 14, 29, 11, 31, 34-37] |  |  |  |
|  |  | Effective utilizing a variety of information and ability to step back and assess situations | [27, 28, 14, 29, 11, 35-37] |  |  |  |
|  | Engagement in nursing representative organizations’ activities | Participate in nursing professional organization activities and incorporate health policy goals | [7, 27, 28, 11, 14, 31, 33-37] |  |  |  |
|  |  | Recognizing the importance of organizing nursing associations and a unified nursing voice | [28, 11, 33, 34, 35, 37] |  |  |  |
|  |  | Maximizing individual strengths within collectives | [28, 11, 34, 35, 37] |  |  |  |
|  |  | Recognizing the need for individual contributions for collective action | [28, 11, 34, 35, 37] |  |  |  |
|  |  | Perception of nursing organizational roles of nurses | [28, 11, 34, 35, 37] |  |  |  |
|  | Participation in political activities or policy intervention | Activating in political participation or policy advocacy of nurses | [7, 27, 28, 14, 11, 31-34, 37, 38] |  |  |  |
|  |  | Focusing on achievements rather than setbacks in political activities | [28, 31, 34, 37] |  |  |  |
|  |  | Enhancing passion, commitment, and political leadership to political engagement or policy intervention | [27, 28, 14, 31, 33-36] |  |  |  |
|  |  | Acknowledgment of small gains amidst political obstacles | [28, 11, 34, 35, 37] |  |  |  |
|  |  | Gaining political experience through public exposure by participating in a variety of political activities | [28, 11, 31, 35] |  |  |  |
|  |  | Recognition of nurses' political influence through participation in political activities | [28, 14, 11, 31, 33, 34, 35, 37] |  |  |  |
|  |  | Importance of initial experiences: Peer skills, such as building and maintaining a strong network of contacts | [27, 28, 14, 11, 31, 34, 35, 37] |  |  |  |
|  |  | Becoming more adaptable in different political healthcare environments | [28, 33, 34, 35, 37] |  |  |  |
|  |  | Becoming a sustainable political nurse activist | [27, 28, 14, 11, 34, 35, 37] |  |  |  |
| **Organizational factors** | | | |  |  | [] |
| Innovating organizational environments | Improvement of nursing work environments | Improving the shift work environment for nurses | [28, 11, 34, 37] |  |  |  |
|  |  | Recruitment of insufficient nursing staff | [28, 11, 34, 37] |  |  |  |
|  |  | Providing appropriate compensation according to nursing work | [28, 34, 37] |  |  |  |
|  | Postering supportive organizational culture | Building an inclusive and supportive organizational culture | [28, 10, 34, 37] |  |  |  |
|  |  | Respecting nurses and moving to participatory management | [28, 10, 34, 37] |  |  |  |
|  |  | Providing resources and support for political participation | [28, 10, 11, 34, 37] |  |  |  |
|  |  | Creating a supportive environment for generational differences | [28] |  |  |  |
| **Professional factors** | | | |  |  |  |
| Enhancing political nursing education | Development and operation of systematic nursing political education curriculum | Developing a political empowerment curriculum and operating theoretical training program to empower nurses | [7, 26, 27, 28, 29, 11, 34, 37] |  |  |  |
|  |  | Motivating nursing students' political awareness and policy engagement | [26, 27, 28, 29, 11, 31, 34, 37] |  |  |  |
|  |  | Cultivating political knowledge (political processes and healthcare system) and leadership of students and nurses | [26, 27, 28, 14, 29, 11, 31, 33-37] |  |  |  |
|  |  | Instructing about historical perspectives on nursing and politics | [26, 27, 28, 14, 29, 11, 33, 34, 36, 37] |  |  |  |
|  |  | Operating of formal/informal learning and engagement for having critical thinking and problems solving skills | [26, 27, 28, 14, 29, 11, 31, 34, 36, 37] |  |  |  |
|  |  | Early life experiences and education | [38] |  |  |  |
|  |  | Promoting to deal with the public, communication (written, verbal, listening, and presentation), and interpersonal skills | [26, 27, 28, 14, 29, 11, 31, 34, 36, 37] |  |  |  |
|  |  | Operating of legislative learning and enhancing policy competence | [26, 27, 28, 14, 29, 11, 33, 34, 36, 37] |  |  |  |
|  | Activating political nursing education for nurturing nursing political activists | Operating training program to strengthen nurses' political skills | [7, 26, 27, 28, 29, 11, 31, 34, 37] |  |  |  |
|  |  | Providing apprenticeship like experience for cultivating political leaders | [26, 28, 29, 11, 34, 37] |  |  |  |
|  |  | Learning through involvement and interacting with people involved in public policy | [26, 28, 29, 11, 31, 34, 37] |  |  |  |
|  |  | Recognizing and cultivating the need for nurse politicians to effectively advance policy | [26, 28, 29, 11, 34, 37] |  |  |  |
|  |  | Supporting for political novices in political settings | [28, 29, 34, 37] |  |  |  |
|  |  | Recognizing the importance of succession planning | [28, 29, 34, 37] |  |  |  |
|  | Development and operation of experiential mentoring and legislative programs | Importance of mentorship and guidance for enhancing political competence | [27, 28, 29, 11, 34, 37] |  |  |  |
|  |  | Activating mentorship for nurturing nursing activists and sharing experiences in health policy | [27, 28, 29, 11, 34, 36, 37] |  |  |  |
|  |  | Importance of mentor relationships: more important than family members or schools | [27, 28, 29, 34, 37, 38] |  |  |  |
|  |  | Importance of experienced and skilled tutors in nursing education | [38] |  |  |  |
|  |  | Providing experiential learning and internship Opportunities such as apprenticeships | [27, 28, 29, 34, 37] |  |  |  |
|  | Strengthening evidence-based research for policy development | Enhancing systematic analysis ability or research to analyse healthcare policy issues | [7, 13, 28, 14, 29, 11, 31, 33-37] |  |  |  |
|  |  | Enhancing analysis or research for developing policy alternatives, proposing operable policy recommendations, and disseminating research findings | [13, 28, 14, 29, 11, 34-37] |  |  |  |
|  |  | Activating research interpretation and operating and utilizing research findings for policy advocacy | [28, 14, 11, 34-37] |  |  |  |
|  |  | Connecting education, practice, and research to enable political engagement and policy intervention | [28, 11, 34, 35, 37] |  |  |  |
| Promoting a supportive system by nursing representative organizations | Establishing a politically supportive system for nurse activists | Motivating nurses' political awareness and policy engagement | [28, 14, 10, 11, 32, 34, 35, 37] |  |  |  |
|  |  | Promoting nurse and nursing organization engagement in health policy development | [28, 14, 10, 34, 35, 37] |  |  |  |
|  |  | Building a nursing professional support network | [28, 33-35, 37] |  |  |  |
|  |  | Raising nurses' expectations about politics by nurse representative organizations | [28, 34, 35, 37] |  |  |  |
|  |  | Increasing the sustainability of nurses' public health advocacy efforts | [28, 10, 11, 34, 35, 37] |  |  |  |
|  |  | Building a support system for nurses' long-term political engagement | [28, 10, 11, 34, 35, 37] |  |  |  |
|  |  | Strengthening professional organizations' support for nurses | [28, 10, 34, 35, 37] |  |  |  |
|  |  | Organizing professional associations' support for nurse political activists | [28, 34, 35, 37] |  |  |  |
|  |  | Accessing to political resources and guidance | [28] |  |  |  |
|  |  | Ongoing training with support from nursing organizations builds confidence in political engagement | [28, 10, 11, 34, 35, 37] |  |  |  |
|  |  | Ongoing support and encouragement of member engagement | [28, 10, 34, 35, 37] |  |  |  |
|  |  | Building collective impact with nursing organization-centric cohesion | [28, 11, 34, 35, 37] |  |  |  |
|  | Enhancing cohesiveness in nursing representative organizations | Involvement and interacting with nurses engaged in public policy: Necessity of individual contributions for collective action | [27, 13, 28, 11, 32, 34-37] |  |  |  |
|  |  | Collective effort and external support by nursing representative organizations for collective power | [27, 13, 28, 34-37] |  |  |  |
|  |  | Influencing collective nursing power through nursing representations: Value of collective action | [27, 13, 28, 34, 35, 37] |  |  |  |
| Activating nursing organizations’ political activities | Building political networking as a source of power | Identifying formal or informal network in political arena and legislators for public advocacy and nurses’ right | [27, 13, 28, 14, 29, 11, 33-37] |  |  |  |
|  |  | Building networks for health policy influence and value of ongoing relationship maintenance | [7, 27, 13, 28, 14, 29, 11, 32, 33-37, 38] |  |  |  |
|  |  | Continuous networking with political and policy officials to gain political influence | [27, 13, 28, 14, 29, 11, 33-37] |  |  |  |
|  |  | Collaboration for shared political goals with interest groups | [28, 29, 11, 31, 34-37] |  |  |  |
|  |  | Collaboration for shared political goals with other healthcare profession or interdisciplinary | [28, 11, 31, 33-37] |  |  |  |
|  |  | Collaboration for shared political goals with nongovernmental organization | [28, 11, 31, 33-37] |  |  |  |
|  |  | Working with diverse social organizations to foster collaborative change to improve healthcare policy | [28, 29, 11, 34-37] |  |  |  |
|  |  | Overcoming special interest conflicts in healthcare system: Coalitions for policy issues | [28, 14, 11, 34, 35, 37] |  |  |  |
|  | Persuasion using effective communication in the political arena | Developing interpersonal skills for enact a nursing perspective and persuading legislators and politicians | [28, 14, 29, 11, 33-37] |  |  |  |
|  |  | Developing effective communication skills in the political arena such as listening, writing and effective communication skills | [7, 28, 14, 29, 11, 33-37] |  |  |  |
|  |  | Effective political interactions: Working relationship and collaboration (teamwork) | [28, 14, 29, 11, 33-37] |  |  |  |
|  |  | Gaining the attention of politicians through persuasion | [7, 28, 11, 33-35, 37] |  |  |  |
|  |  | Utilizing social trust, position, and the nursing professionalism with legislators and the public | [28, 11, 34, 35, 37] |  |  |  |
|  |  | Enhancing political interaction with persuasion and perseverance based on fact | [28, 11, 31, 33-35, 37] |  |  |  |
|  | Enhancing various political activities through developing networks | Building social solidarity with diverse organizations through networking | [13, 28, 14, 29, 11, 34-37] |  |  |  |
|  |  | Engaging in political activism and healthcare policy interventions through the building social solidarity | [7, 13, 28, 14, 29, 11, 34-37] |  |  |  |
|  |  | Enhancing nursing representation’ political activities and creating opportunities | [28, 11, 34, 35, 37] |  |  |  |
|  |  | Expanding the social influence of the nursing profession | [7, 28, 14, 11, 34, 35, 37] |  |  |  |
|  | Formation of social opinion using media | Accumulating political participation experience using various social media network | [28, 14, 11, 34-37] |  |  |  |
|  |  | Overcoming traditional stereotypes about the nursing profession | [28, 11, 34, 35, 37] |  |  |  |
|  |  | Promoting nursing autonomy or public awareness of nursing contributions | [28, 11, 34, 36, 37] |  |  |  |
|  |  | Activating media engagement to shape public perception and sentiment | [7, 28, 11, 13, 33, 34, 37] |  |  |  |
|  |  | Validating and empathizing with public perceptions | [28, 11, 34, 36, 37, 38] |  |  |  |
|  |  | Raising public awareness and advocate for policy change by various media | [28, 11, 33, 34, 37, 38] |  |  |  |
|  |  | Expanding the nursing profession's social advocacy to increase political influence by various media | [28, 11, 34, 36, 37] |  |  |  |
|  |  | Recognizing and utilizing the role of social media in skill development and networking | [28, 11, 33, 34, 37] |  |  |  |
|  |  | Utilization of professional association's social media platform | [28, 11, 34, 36, 37] |  |  |  |
| Enhancing nursing representative organizations’ intervention in healthcare policy reform | Development of healthcare policy reform alternatives | Identifying creative and innovative advocacy strategies in the development of health care policy | [27, 13, 28, 14, 29, 11, 33-37] |  |  |  |
|  |  | Developing evidence-based strategic policy alternatives and formulation | [7, 11, 13, 28, 29, 11, 32, 34, 36, 37] |  |  |  |
|  |  | Increasing nurses' desire to have a voice in policy development | [28, 14, 29, 11, 34, 35, 37] |  |  |  |
|  | Lobbying and petitioning policymakers to reflect the nursing perspective | Building relationships with political lobbyists and lobbying to include nursing's perspective in health policy | [13, 28, 14, 29, 11, 34, 35, 37] |  |  |  |
|  |  | Actively participating in activities supported by lobbyists | [7, 28, 11, 34, 35, 37, 3] |  |  |  |
|  | Participating in healthcare policy-making process | Understanding broader nursing practice, the impact of health policy, and healthcare policy processes | [27, 28, 10, 11, 31, 33-37] |  |  |  |
|  |  | Developing policy alternatives to improve public health and nurses’ right | [7, 27, 28, 14, 10, 11, 33-37] |  |  |  |
|  |  | Analysing health care issues from an inclusive perspective based on critical thinking and problem-solving | [28, 14, 10, 11, 31-38] |  |  |  |
|  |  | Building engagement with political candidates for policy implementation | [27, 28, 14, 11, 10, 34, 35, 37] |  |  |  |
|  |  | Careers in government or government agencies or asserting organizational standpoints in policy forums/seminars | [28, 10, 35-37] |  |  |  |
|  |  | Utilizing nursing's credibility with legislators and the public | [28, 14, 34, 35, 37] |  |  |  |
|  |  | Applying the nursing process or skills to politics or policy intervention | [7, 28, 29, 11, 31, 35] |  |  |  |
|  |  | Accumulating experience in health care system and policy reform | [27, 28, 14, 29, 11, 34-37] |  |  |  |
|  | Implementing and ongoing monitoring of proposed policy reform legislation | Active engagement in the legal or policy-making process: Initiative and proactivity in healthcare setting | [27, 13, 28, 14, 29, 10, 11, 31, 33-37] |  |  |  |
|  |  | Active participating in the community, government, National Assembly, and policy committees for reflecting nursing perspective: Leading change initiatives | [11, 35] |  |  |  |
|  |  | Activities to prevent policy distortion through monitoring of the implementation process of reformative health care policies | [11, 35] |  |  |  |
