# Supplementary material for: The Barriers and Facilitators Influencing Nurses' Political Participation or Healthcare Policy Intervention: A Systematic Review and Qualitative Meta-Synthesis
Source: J Nurs Manag. 2024 Jun 28;2024:2606855. doi: 10.1155/2024/2606855 (PMC11919103; doi:10.1155/2024/2606855)
Supplement: Supplementary Materials — include seven files that provide further information about search strategies, excluded articles based on the full-text review, the PRISMA 2020 checklist, a list of the selected articles for analysis, the findings (barriers and facilitators), the GRADE CERQual assessment, and the eMERGEe reporting result. [file 2606855.f1.zip › 6_SYNT~1.DOC]

**Supplementary table 6. Synthesis finding assessment_ GRADE CERQual evidence profile.**

The reliability of the qualitative evidence synthesis findings was tested using the GRADE-CERQual assessment. This approach facilitated the assessment of the reliability of the results derived through the synthesis of qualitative research.

| **Summary of findings** | **Methodological limitation** | **Coherence** | **Adequacy** | **Relevance** | **CERQual assessment of confidence in the evidence** |
| --- | --- | --- | --- | --- | --- |
| Most nurse activists who participated in the study stated that the “lack of political interest of nurses” was one of the personal barriers to political participation [7, 11, 13, 14, 26-28, 34, 37, 38]. In particular, it was reported that nurses’ interest in political participation varies depending on nurses’ gap of political beliefs and values [7, 11, 13, 14, 26-28, 34, 37, 38], and it tends to be low due to the lack of political efficacy of nurses’ political participation influencing the field of healthcare policy [7, 10, 11, 14, 26, 28, 34, 37, 38]. | Minor methodological limitations: 1/11 study with unclear evidence of reflexivity | No concerns about coherence of data | No concerns about adequacy of data (11 studies) | No concerns about relevance of data (11 studies) | High confidence:  The 1 studies of moderate quality, with minor methodological limitations, high coherence, high relevance, and high concerns about adequacy of data. |
| Finding 2: Nurse activists reported that most nurses’ “lack of political competence” [7, 10, 11, 13, 14, 27-29, 34, 37] is due to the lack of political knowledge and information [7, 10, 11, 13, 14, 27-29, 34, 37] regarding political processes, policy development, and advocacy strategies, as well as lack of political skills such as communication skills and political networking [7, 10, 11, 13, 14, 28, 29, 34, 37]. Additionally, although healthcare policy interventions were feasible to maximize the strengths of individual nurses through collective action [35], they face a limitation that nurses’ lack of participation in activities of nursing representative organizations diminishes their collective voice in policy-making [28, 31, 34, 37]. The lack of political participation reflects nurses’ minimal involvement in political activities, such as advocacy, lobbying, and interaction with legislators or policymakers [7, 10, 11, 14, 26, 28, 29, 34, 37, 38]. In addition, nurses’ lack of awareness of the policy process, without a clear grasp, may make them ill-equipped to contribute to policy discussions or effectively advocate for policy changes [7, 10, 11, 14, 26. 28, 29, 34, 37, 38]. | Minor methodological limitations: 1/13 study with unclear evidence of reflexivity | No concerns about coherence of data | No concerns about adequacy of data (13 studies) | No concerns about relevance of data (13 studies) | High confidence:  The 1 studies of moderate quality, with minor methodological limitations, high coherence, high relevance, and high concerns about adequacy of data. |
| Finding 3: Poor working environments [10, 11, 26, 28, 32, 34, 37], as a sub-theme of “nurses’ working environment constraints,” are a significant barrier to nurses’ political participation by limiting their capacity and motivation to engage in activities beyond their immediate clinical responsibilities [10, 11, 26, 28, 32, 34, 37]. A challenging work environment characterized by high stress, shift work, long hours, insufficient staffing levels, and a lack of resources leaves nurses physically and emotionally drained, reducing their availability and energy for political advocacy or policymaking activities. Furthermore, poor working environments create time and resource constraints for nurses, which act as a barrier to political participation because it requires time, resources, and sacrifice at an individual level [10, 11, 14, 26, 28, 34, 37]. | Minor methodological limitations: 1/8 study with unclear evidence of reflexivity | No concerns about coherence of data | No concerns about adequacy of data (8 studies) | No concerns about relevance of data (8 studies) | High confidence:  The 1 studies of moderate quality, with minor methodological limitations, high coherence, high relevance, and high concerns about adequacy of data. |
| Finding 4: The sub-theme of nepotism and favoritism within the organization under the theme “barriers to organizational culture” refers to practices within healthcare and nursing education institutions where opportunities for involvement in policy-making are unfairly allocated based on personal relationships rather than merit, skills, or professional qualifications [26, 28, 34, 35, 37]. This creates significant barriers for nurses who seek to participate in political and policy intervention activities but find themselves excluded or marginalized because of these biased practices. Furthermore, the hierarchical and structural organizational culture within healthcare institutions, with rigidly stratified and formal organizational structures, may impede nurses' political participation [10, 26, 28, 30, 32, 34, 37, 38]. Meanwhile, “generational differences among nurses may act as barriers to cohesive action and participation in policy intervention due to the varying levels of interest, engagement, and methods of communication preferred by different generations [27, 28]. | No methodological limitations: 10/10 studies with clear evidence of reflexivity | No concerns about coherence of data | No concerns about adequacy of data (10 studies) | No concerns about relevance of data (10 studies) | High confidence:  No moderate methodological limitations, high coherence, high relevance, and high concerns about adequacy of data. |
| Finding 5: Relating to the “professional stereotypes of the nursing profession," the sub-theme of undervaluation of nurses’ expertise reflects a systemic issue in which nurses’ knowledge and contributions are not fully valued in society or the political arena [7, 10, 14, 26, 28-30, 34, 37]. In addition, the nursing profession, historically and predominantly female, faces gender biases that further compound the issue of undervaluation [13, 26, 28, 30, 32, 34, 37, 38]. In a world where men are primarily responsible for policy-making roles, the gender limitations of women in nursing professions also negatively affect performing policy-making functions [13, 26, 28, 30, 32, 34, 37, 38]. | Minor methodological limitations: 1/12 study with unclear evidence of reflexivity | No concerns about coherence of data | No concerns about adequacy of data (12 studies) | No concerns about relevance of data (12 studies) | High confidence:  The 1 studies of moderate quality, with minor methodological limitations, high coherence, high relevance, and high concerns about adequacy of data. |
| Finding 6: Most nurse activists pointed out that the “lack of political nursing education” are serious professional barriers that undermine nurses’ political participation [7, 10, 11 ,14, 26-31, 34, 35, 37]. In this regard, most nurse activists pointed out insufficient education and training in political competence [7, 10, 11, 14, 26-31, 34, 35, 37] and a lack of mentorship and legislative internship [7, 10, 11, 26, 28, 29, 34, 37], especially political mentorship, which is crucial for developing nurses’ political skills and policy competence [28, 33, 34, 37]. Without the guidance and support of an experienced mentor, aspiring nurse activists may struggle to find a pathway to political engagement, develop the necessary confidence and skills, and navigate the challenges of policy advocacy and political participation [7, 10, 11, 26, 28, 29, 34, 37]. | Minor methodological limitations: 2/13 study with unclear evidence of reflexivity | No concerns about coherence of data | No concerns about adequacy of data (13 studies) | No concerns about relevance of data (13 studies) | High confidence:  The 2 studies of moderate quality, with minor methodological limitations, high coherence, high relevance, and high concerns about adequacy of data. |
| Finding 7: The sub-themes of insufficient advocacy efforts for nurses [7, 28, 34, 35, 37], deficiency of political resources and minimal encouragement for political participation [10, 28, 29, 34, 37] under the theme “limited of support for nurses by the nursing representative organizations” were also reported as professional barriers. Nurse activists pointed out that although nursing representative organizations are making efforts to improve the poor working environment of nurses and defend the public's right to health, it is still insufficient, and improvements are needed [10, 28, 29, 34, 37]. Additionally, without encouragement and support for political participation from representative organizations, nurses may not perceive political participation as a professional responsibility [35]. | Minor methodological limitations: 1/7 study with unclear evidence of reflexivity | No concerns about coherence of data | No concerns about adequacy of data (7 studies) | No concerns about relevance of data (7 studies) | High confidence:  The 1 studies of moderate quality, with minor methodological limitations, high coherence, high relevance, and high concerns about adequacy of data. |
| Finding 8: Most nurse activists stated the “lack of political power of nursing representative organizations [10, 11, 14, 26, 28, 34, 35, 37].” Relating to this, the specific barriers are not only a lack of political network-building and communication skills and conflict management capabilities among interest groups [10, 11, 28, 34, 35, 37] but also interaction barriers with legislators and difficulty articulating nursing perspectives [10, 11, 14, 26, 28, 34, 35, 37], and insufficient intervention in policy decision-making processes [7, 10, 11, 14, 26, 28, 29, 32, 34, 35, 37, 38]. | Minor methodological limitations: 1/12 study with unclear evidence of reflexivity | No concerns about coherence of data | No concerns about adequacy of data (12 studies) | No concerns about relevance of data (12 studies) | High confidence:  The 1 studies of moderate quality, with minor methodological limitations, high coherence, high relevance, and high concerns about adequacy of data. |
| Finding 9: Increasing nurses’ “recognition of social responsibilities” through awareness of healthcare problems [7, 10, 11, 14, 27-38] and enhancing nursing professional values [7, 11, 28, 30-37, 38] is a factor in encouraging a commitment to political participation or policy intervention among nurses. | Moderate methodological limitations: 4/16 studies with unclear evidence of reflexivity | No concerns about coherence of data | No concerns about adequacy of data (16 studies) | No concerns about relevance of data (16 studies) | Moderate confidence:  The 4 studies of moderate quality, with moderate methodological limitations, high coherence, high relevance, and high concerns about adequacy of data. |
| Finding 10: Most nurse activists emphasized “enhancing nurses’ political competence” [7, 11, 13, 14, 27-29, 31, 32-37]. They tried strengthening inner political power by accumulating political knowledge, information, and skills on public health problems and policymaking [7, 11, 13, 14, 27-29, 31, 32-37]. They also began to lead healthcare policy reform based on internal and external power accumulated through active political activities, that is, political competence [34, 37]. During this process, they actively participated in political activities within the nursing profession [7, 11, 13, 14, 27-29, 31, 32-37]. Therefore, it is important to enhance political competence and encourage participation in political activities or policy interventions [7, 11, 14, 27, 28, 31-38] through the accumulation of political knowledge, information, and skills [7, 11, 13, 14, 27-29, 31, 32-37] and engagement in nursing representative organization’ activities [7, 11, 14, 27-29, 31, 33-37]. | Moderate methodological limitations: 5/15 studies with unclear evidence of reflexivity | No concerns about coherence of data | No concerns about adequacy of data (15 studies) | No concerns about relevance of data (12 studies) | Moderate confidence:  The 5 studies of moderate quality, with moderate methodological limitations, high coherence, high relevance, and high concerns about adequacy of data |
| Finding 11. To improve poor working environments, which is one of the organizational barriers, efforts are needed to ensure adequate staffing levels, improve nursing working environments [11, 28, 34, 37], and provide adequate compensation to nurses [27, 28]. Also, by improving the job satisfaction and morale of nurses through “innovating organizational environments,” nurses could be encouraged to participate more actively in political activities [10, 11, 28, 34, 37]. In addition, to overcome the limitations of organizational culture, such as favoritism, hierarchical and structured organizational culture, and generational differences, nursing organizations and healthcare institutions need to foster a culture of collaboration and inclusivity [27, 28]. Further, encouraging open communication, shared decision-making, and flattening hierarchical structures are necessary for postering supportive organizational culture, which could promote active policy debate and political competence of nurses [10, 11, 28, 34, 37]. | Minor methodological limitations: 1/6 study with unclear evidence of reflexivity | No concerns about coherence of data | No concerns about adequacy of data (6 studies) | No concerns about relevance of data (6 studies) | High confidence:  The 1 studies of moderate quality, with minor methodological limitations, high coherence, high relevance, and high concerns about adequacy of data. |
| Finding 12: Among the professional factors promoting nurses’ political participation, the most frequently stated by nurse activists was “enhancing political nursing education” [7, 11, 14, 26, 28, 29, 31, 33-37], and they reported that education promotes nurses’ professional values such as identity, vision, passion, and confidence [11, 28, 31, 33-37]. Most of them stated that they obtained political knowledge, skills, and information through work or political experience more than formal nursing education due to the lack of formal education [11, 13, 26, 27, 28, 34-37]. Accordingly, development and operation of systematic nursing political education curriculum, a variety of formal and informal education, is required for nurses [7, 11, 14, 26, 28, 29, 31, 33-37]. Additionally, the developing and operating of experiential mentoring and legislative programs is effective in activating political nursing education and nurturing nursing political activists [7, 11, 26-29, 31, 34, 37]. By providing training in political skills for nurses to improve practical interpersonal skills such as communication, persuasion, and negotiation, it could develop nurses’ political competence [7, 11, 26-29, 31, 34, 37]. Furthermore, strengthening evidence-based research for policy development is critical for reflecting nursing perspectives into healthcare policies [7, 11, 13, 14, 28, 29, 31, 33-37]. | Moderate methodological limitations: 5/14 studies with unclear evidence of reflexivity | No concerns about coherence of data | No concerns about adequacy of data (14 studies) | No concerns about relevance of data (14 studies) | Moderate confidence:  The 5 studies of moderate quality, with moderate methodological limitations, high coherence, high relevance, and high concerns about adequacy of data. |
| Finding 13: Some nurse activists emphasize “promoting a supportive system by nursing representative organizations” [10, 11, 13, 14, 28, 32, 34, 35, 37] through the establishment of a politically supportive system for nursing activists [10, 11, 14, 28, 32, 34, 35, 37]. In addition, to secure political opportunities for nurses, who account for most healthcare professionals, it is crucial to enhance cohesiveness in nursing representative organizations or civic organizations [11, 13, 27, 28, 32, 34-37]. This makes it possible contributing to a unified social voice of the nursing profession [35]. | Minor methodological limitations: 3/11 studies with unclear evidence of reflexivity | No concerns about coherence of data | No concerns about adequacy of data (11 studies) | No concerns about relevance of data (11 studies) | High confidence  The 3 studies of moderate quality, with minor methodological limitations, high coherence, high relevance, and high concerns about adequacy of data. |
| Finding 14: Building political networking as a source of power [7, 11, 13, 14, 27-29, 31-38] is most important for “activating nursing organizations’ political activities [7, 11, 13, 14, 27-29, 31-38].” Networking is a strategy that influences health policy and contributes to expanding nurses’ political participation by securing a basis for communication and support from politicians, including members of the National Assembly [11, 35]. In addition, establishing social solidarity with various patient groups, healthcare representative organizations, and municipal government organizations is effective in exercising political influence by nursing organizations’ enhancing various political activities through developing networks [7, 11,13,14,28,29,32,34-38]. Mainly, it is necessary to be familiar with the language and laws used by politicians and to conduct persuasion using effective communication in the political arena, such as writing, listening, presentation, and conflict resolution skills with perseverance [7,11, 14, 28, 29, 31, 33-37]. In addition, one of the effective facilitating strategies is the formation of social opinion using media [7, 11, 14, 28, 34-37]. | Moderate methodological limitations: 5/15 studies with unclear evidence of reflexivity | No concerns about coherence of data | No concerns about adequacy of data (15 studies) | No concerns about relevance of data (15 studies) | Moderate confidence:  The 5 studies of moderate quality, with moderate methodological limitations, high coherence, high relevance, and high concerns about adequacy of data. |
| Finding 15: Relating to “enhancing nursing representative organization’s intervention in healthcare policy reform [7, 11, 13, 14, 27-29, 31-38],” nurse activists sought policy intervention by identifying issues and analyzing healthcare problems, then development of healthcare policy reform alternatives [7, 11, 13, 14, 27-29, 32-37] and lobbying and petitioning policymakers to reflect the nursing perspective [7, 11, 13, 14, 28, 29, 34, 35, 37, 38]. Nurse activists emphasized that healthcare policy reform alternatives should be developed first to activate healthcare policy intervention [7, 11, 13, 14, 27-29, 32-37]. Thus, it is proactively necessary to understand issues and policymaking processes to predict current and future healthcare problems [7, 11, 13, 14, 27-29, 32-37]. Then, lobby or petition to persuade legislators, elected officials, and politicians [7, 11, 13, 14, 28, 29, 34, 35, 37, 38] and those experiences create opportunities for participating in the healthcare policy-making process as members of the government policy deliberation committee, members of parliament, and city councilors [7, 10, 11, 14, 27-29, 31-38]. This ultimately contributes to the realization of the proposed healthcare policy reform legislation reflecting the nursing perspective [11, 13, 29, 34, 35, 37]. In this process, nurses must implement and monitor the proposed policy reform legislation to properly implement the reform policies or bills without distortion in the field [11, 35]. | Moderate methodological limitations: 5/16 studies with unclear evidence of reflexivity | No concerns about coherence of data | No concerns about adequacy of data (16 studies) | No concerns about relevance of data (16 studies) | Moderate confidence:  The 5 studies of moderate quality, with moderate methodological limitations, high coherence, high relevance, and high concerns about adequacy of data. |
