# Supplementary material for: The Barriers and Facilitators Influencing Nurses' Political Participation or Healthcare Policy Intervention: A Systematic Review and Qualitative Meta-Synthesis
Source: J Nurs Manag. 2024 Jun 28;2024:2606855. doi: 10.1155/2024/2606855 (PMC11919103; doi:10.1155/2024/2606855)
Supplement: Supplementary Materials — include seven files that provide further information about search strategies, excluded articles based on the full-text review, the PRISMA 2020 checklist, a list of the selected articles for analysis, the findings (barriers and facilitators), the GRADE CERQual assessment, and the eMERGEe reporting result. [file 2606855.f1.zip › 7_EMER~1.DOC]

**Supplementary table 7. eMERGe reporting criteria for meta-ethnography**

The result of the eMERGe reporting criteria for meta-ethnography is in (Supplementary table 7).

| **No.** | **Criteria Headings** | **Reporting Criteria** |
| --- | --- | --- |
| Phase 1—Selecting meta-ethnography and getting started | | |
| *Introduction* | | |
| 1 | Rationale and context for the meta-ethnography | Describe the gap in research or knowledge to be filled by the meta-ethnography, and the wider context of the meta-ethnography.  Introduction – page 2, 3 |
| 2 | Aim(s) of the meta-ethnography | Describe the meta-ethnography aim(s)  Introduction – page 2, 3 |
| 3 | Focus of the meta-ethnography | Describe the meta-ethnography review question(s) (or objectives)  Introduction – page 3 |
| 4 | Rationale for using meta-ethnography | Explain why meta-ethnography was considered the most appropriate qualitative synthesis methodology  Introduction – page 2, 3 |
| Phase 2—Deciding what is relevant | | |
| *Methods* | | |
| 5 | Search strategy | Describe the rationale for the literature search strategy  Section 2.2 – page 3, 4 |
| 6 | Search processes | Describe how the literature searching was carried out and by whom  Section 2.2 – page 4 |
| 7 | Selecting primary studies | Describe the process of study screening and selection, and who was involved  Section 2.3.1 – page 4  Section 2.3.2 – page 5  Section 2.3.3 – page 6 |
| *Findings* | | |
| 8 | Outcome of study selection | Describe the results of study searches and screening  Section 2.3.2 – page 7-8 |
| Phase 3—Reading included studies | | |
| *Methods* | | |
| 9 | Reading and data extraction approach | Describe the reading and data extraction method and processes  Section 2.4.1 – page 11  Section 2.4.2 – page 12 |
| *Findings* | | |
| 10 | Presenting characteristics of included studies | Describe characteristics of the included studies  Methods: Section 2.3.2 – page 8-11 |
| Phase 4—Determining how studies are related | | |
| *Methods* | | |
| 11 | Process for determining how studies are related | Describe the methods and processes for determining how the included studies are related: - Which aspects of studies were compared AND - How the studies were compared  Section 2.4.2 – page 12 |
| *Findings* | | |
| 12 | Outcome of relating studies | Describe how studies relate to each other  Section 3 – Synthesis Finding: page 13-20 |
| Phase 5—Translating studies into one another | | |
| *Methods* | | |
| 13 | Process of translating studies | Describe the methods of translation**:** - Describe steps taken to preserve the context and meaning of the relationships between concepts within and across studies- Describe how the reciprocal and refutational translations were conducted- Describe how potential alternative interpretations or explanations were considered in the translations  Section 2.4.2 – page 12 |
| *Findings* | | |
| 14 | Outcome of translation | Describe the interpretive findings of the translation.  Section 3 – Synthesis Finding: page 13-20 |
| Phase 6—Synthesizing translations | | |
| *Methods* | | |
| 15 | Synthesis process | Describe the methods used to develop overarching concepts (“synthesised translations”)Describe how potential alternative interpretations or explanations were considered in the synthesis  Section 2.4.2 – page 12 |
| *Findings* | | |
| 16 | Outcome of synthesis process | Describe the new theory, conceptual framework, model, configuration, or interpretation of data developed from the synthesis  Section 3 – Synthesis Finding: page 13-20  Section 4 – Discussion: page 20-22 |
| Phase 7—Expressing the synthesis | | |
| *Discussion* | | |
| 17 | Summary of findings | Summarize the main interpretive findings of the translation and synthesis and compare them to existing literature  Section 4 – Discussion: page 20-22 |
| 18 | Strengths, limitations, and reflexivity | Reflect on and describe the strengths and limitations of the synthesis: - Methodological aspects—for example, describe how the synthesis findings were influenced by the nature of the included studies and how the meta-ethnography was conducted.- Reflexivity—for example, the impact of the research team on the synthesis findings  Section 4 – Discussion: page 22 |
| 19 | Recommendations and conclusions | Describe the implications of the synthesis  Section 5 – Conclusion: page 22-23 |
